# Determinants of *Staphylococcus aureus* carriage in the developing infant nasal microbiome

**DOI:** 10.1186/s13059-020-02209-7

**Published:** 2020-12-11

**Authors:** Emma K. Accorsi, Eric A. Franzosa, Tiffany Hsu, Regina Joice Cordy, Ayala Maayan-Metzger, Hanaa Jaber, Aylana Reiss-Mandel, Madeleine Kline, Casey DuLong, Marc Lipsitch, Gili Regev-Yochay, Curtis Huttenhower

**Affiliations:** 1 Harvard T. H. Chan School of Public Health, 677 Huntington Avenue, Boston, MA 02115 USA; 2 Broad Institute, 415 Main St., Cambridge, MA 02142 USA; 3 Wake Forest University, 1834 Wake Forest Rd., Winston-Salem, NC 27109 USA; 4 Sackler Faculty of Medicine, Tel Aviv University, 69978 Ramat Aviv, Tel Aviv, Israel; 5 Sheba Medical Center, Derech Sheba 2, Ramat Gan, Israel; 6 Harvard Medical School, 25 Shattuck St., Boston, MA 02115 USA

**Keywords:** Nasal, Microbiome, *S. aureus*, Infant, Development, Carriage, Shotgun metagenomics, Longitudinal

## Abstract

**Background:**

*Staphylococcus aureus* is a leading cause of healthcare- and community-associated infections and can be difficult to treat due to antimicrobial resistance. About 30% of individuals carry *S. aureus* asymptomatically in their nares, a risk factor for later infection, and interactions with other species in the nasal microbiome likely modulate its carriage. It is thus important to identify ecological or functional genetic elements within the maternal or infant nasal microbiomes that influence *S. aureus* acquisition and retention in early life.

**Results:**

We recruited 36 mother-infant pairs and profiled a subset of monthly longitudinal nasal samples from the first year after birth using shotgun metagenomic sequencing. The infant nasal microbiome is highly variable, particularly within the first 2 months. It is weakly influenced by maternal nasal microbiome composition, but primarily shaped by developmental and external factors, such as daycare. Infants display distinctive patterns of *S. aureus* carriage, positively associated with *Acinetobacter* species, *Streptococcus parasanguinis*, *Streptococcus salivarius*, and *Veillonella* species and inversely associated with maternal *Dolosigranulum pigrum*. Furthermore, we identify a gene family, likely acting as a taxonomic marker for an unclassified species, that is significantly anti-correlated with *S. aureus* in infants and mothers. In gene content-based strain profiling, infant *S. aureus* strains are more similar to maternal strains.

**Conclusions:**

This improved understanding of *S. aureus* colonization is an important first step toward the development of novel, ecological therapies for controlling *S. aureus* carriage.

**Supplementary Information:**

The online version contains supplementary material available at 10.1186/s13059-020-02209-7.

## Background

*Staphylococcus aureus* is a leading cause of healthcare-associated infections among infants [1] and adults [2], and it is the most common cause of hospital- and community-acquired skin and soft tissue infections (SSTIs) [3]. Antibiotic resistance, in particular, to β-lactams (e.g., methicillin-resistant *S. aureus* (MRSA)), makes the treatment of these infections difficult and expensive [4]. *S. aureus* is responsible for approximately 119,000 bloodstream infections and 20,000 deaths per year in the USA alone [5]. Nasal colonization by *S. aureus* increases the risk of SSTIs [6] and is a risk factor for bacteremia, as 80% of bloodstream infections are caused by the same strain found in the individual’s nose [7–10]. Additionally, colonization allows for transmission to new hosts and selection for new, possibly detrimental microbial traits, such as antibiotic resistance [11]. At any one time, approximately 30% of individuals carry this species asymptomatically in their nares [12], and over time, individuals display three carriage patterns: persistent (~ 20%), transient (~ 60%), and never (~ 20%) carriers [13–16]. It is unknown what causes these differences between individuals [13–16], and research in infants is especially scarce [17].

Previous studies suggest that both *S. aureus* carriage and nasal microbiome composition are primarily determined by environmental rather than host genetic factors [18, 19]. Co-occurring nasal species can provide resistance to *S. aureus* colonization through competition for nutrients and binding sites [20, 21], such as *Corynebacterium* strain Co304 which is believed to outcompete *S. aureus* at binding nasal mucin [21], or through the induction of a host immune response [22–24], such as the targeting of *S. aureus* by a cross-reactive antibody triggered against *Streptococcus pneumoniae* [23]. Additionally, some nasal species produce antimicrobial molecules that are directly toxic to *S. aureus* [25–30], such as hydrogen peroxide produced by *S. pneumoniae* [29] or the peptide antibiotic lugdunin produced by *Staphylococcus lugdunensis* [30]. While laboratory studies have identified these mechanisms, it is not clear how they translate into the observed population-level trends in *S. aureus* carriage. For instance, although lugdunin is highly effective at killing *S. aureus* and the risk of *S. aureus* colonization is sixfold-lower in those colonized with *S. lugdunensis*, the population prevalence of *S. lugdunensis* (estimated at 9% [30, 31] or 26% [32]) is too low to explain why 80% of individuals only temporarily or never acquire *S. aureus* [30, 33]. Similarly, some *Staphylococcus epidermidis* isolates produce lantibiotics, and many produce an extracellular serine protease Esp that inhibits *S. aureus* colonization [34, 35], but *S. epidermidis* also does not consistently predict *S. aureus* absence [19, 32, 35, 36]. Understanding nasal microbial community structure, ecology, metabolism, and signaling thus holds promise for elucidating patterns of *S. aureus* carriage, but many complementary and competing mechanisms likely contribute to its ultimate colonization pattern [33]. Human population studies using shotgun metagenomic measures that are both detailed and comprehensive are thus needed to characterize nasal microbial communities with a high degree of phylogenetic and functional resolution.

Understanding the relationship between the nasal microbiome and *S. aureus* carriage in early life is especially important because the infant microbiome across the body undergoes dramatic shifts in the first year [37, 38], and infants have also been shown to acquire and lose *S. aureus* in the nares during this time [39, 40]. Prior studies of *S. aureus* carriage have mainly been performed in adults and are not necessarily generalizable to infants, as the infant microbiome differs greatly from that of adults during this early period of rapid development [41, 42]. Infant nasal microbiome colonization may be influenced by many of the same environmental factors as the gut microbiome (e.g., delivery mode, feeding method, antibiotic usage, and siblings [42–44]), but it has been substantially less studied than has the gut [11, 43, 45]. Particularly with the goal of mitigating later *S. aureus* infection risk, additional longitudinal studies in this area can determine whether microbiome changes causally precede *S. aureus* acquisition or loss and shed light on the development of the infant nasal microbiome, which is also broadly involved in later asthma, allergies, and upper respiratory tract infections [44, 46–48].

In this study, we aimed to identify intra-community interactions involved in the modulation of *S. aureus* carriage in vivo and to characterize the development of the early infant nasal microbiome. We examined nasal microbiome development in 36 mother-infant pairs over the first year after birth, combining shotgun metagenomic profiling of longitudinal samples with monthly *S. aureus* culture-based testing. Both methods generally agreed in their prevalence of *S. aureus* carriage (per sample, 43.8% by culture vs. 38% by sequencing) and were used to define subpopulations of early-, late-, and non-*S. aureus* acquirers. In gene content-based strain profiling, infants carried strains of *S. aureus* that were more similar to maternal strains compared to unrelated mothers. Furthermore, we identified a collection of species associated with patterns of *S. aureus* carriage among infants, as well as a possible uncharacterized taxon significantly anticorrelated with *S. aureus* in infants and mothers. With the CDC reporting slowed progress on methicillin-resistant and methicillin-susceptible *S. aureus* infection reduction in hospitals and communities [5], understanding such dynamics of *S. aureus* colonization in the nasal microbiome is an important first step toward the development of safe and mechanistically understood ecological therapies for modulating *S. aureus* carriage.

## Results

### Patterns of *S. aureus* carriage in a mother-infant cohort

**Fig. 1:**
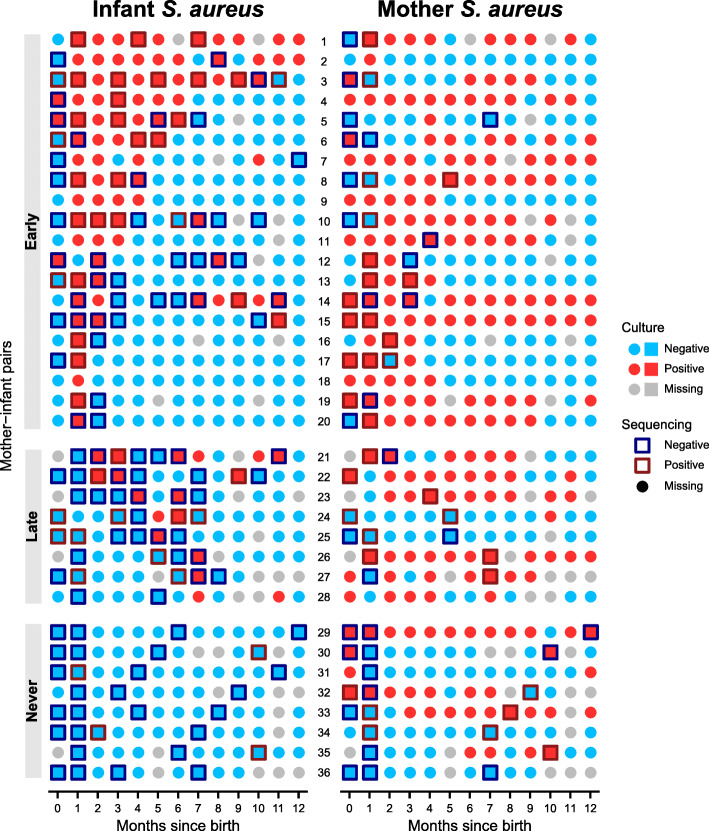
Longitudinal shotgun metagenomics of the nasal microbiome and *Staphylococcus aureus* carriage in 36 mother-infant pairs. Nasal swabs were taken from 36 mother-infant pairs at birth and monthly for the first year after birth. Culture testing for *S. aureus* was performed on all samples to assess carriage. Culture results, red for *S. aureus* positive and blue for *S. aureus* negative, are shown as the icon fill color. A subset of 208 samples was profiled with shotgun metagenomic sequencing and retained after quality control (the “Methods” section) for further analyses. Sequencing is shown as performed only for samples that passed QC procedures. Square icons indicate samples where sequencing was performed, and the outline color, red for *S. aureus* positive and blue for *S. aureus* negative, indicates whether *S. aureus* was identified by sequencing. Infants displayed three unique patterns of *S. aureus* carriage highlighted here and used for sequenced sample prioritization: “Early” (acquisition by month one), “Late” (acquisition after month one, also typically transient), and “Never” (no acquisition during the study period)

We collected monthly longitudinal nasal swab samples from a total of 36 mother-infant pairs recruited through Sheba Medical Center as part of a larger cohort study on *S. aureus* [17], as well as *S. aureus*-negative mothers enrolled specifically for this study (the “Methods” section). A subset of samples was selected for microbial community profiling by culture testing a total of 856 samples from mothers and infants, both throughout the first year after birth. Samples were selected for shotgun metagenomics to include early time points (i.e., soon after delivery) for all subjects, later time points (as representatives) from selected mothers, and time points immediately preceding and following changes in *S. aureus* carriage status as determined by culture test in infants (as well as additional, typically later, representative infant time points in order to assess developmental changes). This resulted in a total of 284 samples profiled metagenomically, with 208 samples passing sample quality control procedures (Fig. 1, the “Methods” section).

During the first year of life, infants displayed distinctive *S. aureus* carriage phenotypes. Based on culture, 20 out of 36 infants (56%) acquired *S. aureus* for the first time at or before month 1 (“early” acquisition), and three of these infants displayed persistent colonization, defined as being positive for at least two thirds of all time points and for at least half of time points between months 6 and 12. Of those remaining, eight infants (22%) never acquired *S. aureus* over the study period (“never” acquisition), and eight infants (22%) acquired *S. aureus* after month 1 but were never persistently colonized (“late” acquisition). Among retained samples, we sequenced 71, 43, and 30 samples from early, late, and never acquirer infants, respectively, as well as 13 samples from persistently colonized infants and 64 samples from mothers (Fig. 1).

Detection of *S. aureus* by culture and sequencing showed moderate agreement; as expected, samples that were positive for *S. aureus* by culture had a higher relative abundance of *S. aureus* by sequencing compared to culture-negative samples (mean 13.53% vs. 1.09%; median 0.45% vs. 0%; Wilcoxon *p* < 10^−7^ across all 208 filtered samples). Since both culture and sequencing have low rates of false positives, we believe disagreement between the two tests arises from the combination of a true positive from one test and a false negative from the other, although the mechanisms causing false negatives differ between culture and sequencing. Previous work found that the sensitivity of culture is highly positively correlated with *S. aureus* absolute abundance, which suggests that culture-based methods alone may miss a significant fraction of *S. aureus* carriage [19], in part due to idiosyncratic mechanisms such as viable but non-culturable biofilm formation [49]. Conversely, any sufficiently rare microbe—including *S. aureus*—can be missed by sequencing of insufficient depth (which in sites such as the nares includes human nucleotides; Additional file 1: Fig. S1). Due to these likely false negatives from both assays, our subsequent analyses included a composite variable for *S. aureus* positivity by culture or sequencing.

### Infant nasal microbiome composition matures over the first year but remains distinct from that of the mothers

**Fig. 2:**
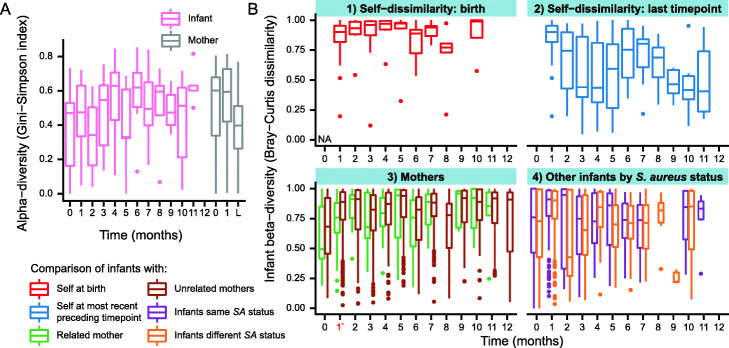
Development of infant nasal microbiome composition over the first year of life. **a** Infant nasal microbiome development showed general ecological patterns similar to those of other body sites. Alpha diversity increased over time in infants, although confidence was lower at later time points due to fewer samples; it decreased slightly at later time points (L) in mothers. Mothers were sequenced at birth, month 1, and one later time point (matched to a later time point for their infant, making this not the same time point for all mothers), which is labeled as “L.” **b** Mean Bray-Curtis dissimilarity per time point comparing (1) infants to themselves at birth, (2) infants to themselves at their most recent preceding time point, (3) infants to related and unrelated mothers, and (4) infants to other infants with the same or different *S. aureus* status at the same time point. In (1), the Bray-Curtis dissimilarity at birth is equal to 0 since we are comparing identical compositions and is labeled as “NA.” The use of the most recently recorded preceding time point for (2) is conservative because we have less frequent sampling at later time points but still find that infants are more self-conserved at these times. *S. aureus* status as positivity by either sequencing or culture is shown here, but there were no group differences if *S. aureus* was defined using other phenotypes. In both graphs, time points with fewer than 5 data points are not displayed (see Additional file 2 for sample sizes), and diversity measures are calculated on average subject taxonomic composition for the mothers (due to the limited sample numbers)

Individual infants’ species composition rapidly diverged from that observed at birth and, over time, was increasingly dissimilar to this early composition (Fig. 2). Controlling for subject, time was significantly associated with infant microbiome composition (PERMANOVA on Bray-Curtis dissimilarity, *p* = 0.001). The rate of change in species composition declined over time and infants became slightly more self-conserved, consistent with stabilization toward a more mature nasal microbiome and somewhat similar to early life gut microbiome development [50] (Fig. 2). However, infant composition remained significantly different from maternal composition across all months except month eight (PERMANOVA on Bray-Curtis dissimilarity, *p* < 0.05), indicating that by the end of the first year, infants still did not have a fully adult nasal microbiome. These findings are consistent with those for the infant skin and gut microbiomes, which are compositionally unstable relative to adults [41, 51] and increase in alpha diversity [41, 51] over the first year, although reported alpha diversity trends in the nasal microbiome of healthy infants are inconsistent [44, 46]. In line with previous research that proposes that nasal microbiome development likely continues after year 1 [42], we find that infants are still significantly different from adults at the latest time points.

Infants were significantly more similar to their own mothers than unrelated mothers at month 1, but not at other time points (Wilcoxon-Mann-Whitney test, *p* = 0.0026); this trend was generally true of early months, although it did not otherwise reach significance (Fig. 2). This supports the hypothesis that maternal influence on nasal microbiome composition mainly occurs at the earliest time points after birth. It is also consistent with studies on the infant gut microbiome, which find greater similarity between vaginally delivered infants and their mothers shortly after birth, but that this similarity diminishes afterwards, often quickly, based on environmental exposures [50, 52–54]. Lastly, the composition of the infant microbiome over time was not related to infant *S. aureus* status for the *S. aureus* phenotypes tested (PERMANOVA on Bray-Curtis dissimilarity, *p* ≥ 0.05) (Fig. 2). This is reassuring given that omnibus testing on the full microbiome captures large differences in composition between samples, which likely would have been identified in earlier (e.g., culture-based) studies if grossly responsible for *S. aureus* phenotypes.

### Species-level and functional drivers of infant *S. aureus* phenotypes

**Fig. 3:**
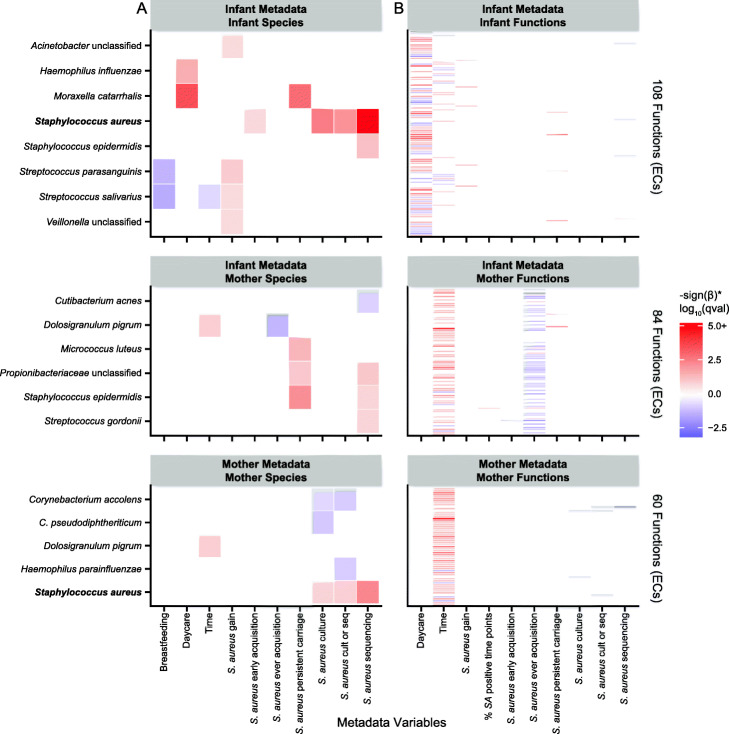
Significant associations between nasal microbiome taxonomic and functional composition and subject phenotypes in feature-wise testing. Significant associations (*q* < 0.25) between individual taxonomic and functional features and phenotypic covariates using a MaAsLin multivariable linear model. **a** Of note, first attendance at daycare (during the preceding month) was an extremely strong determinant of initial microbiome colonization by several taxa (*M. catarrhalis*, *H. influenzae*), and the nasal microbiome exhibited a much weaker time dependence than does the gut during infant development (i.e., few taxa were consistently temporally variable between months 0 and 12). The presence of a number of oral-associated species (i.e., *Streptococcus* and *Veillonella* species) was a mild correlate of *S. aureus* gain. *S. aureus* relative abundance was included as a positive control. **b** After applying a species dominance filter, we found the association of many gene families (grouped by Enzyme Commission (EC) number) in infants with daycare attendance, while many functions in mothers were associated with time and infant “ever” acquisition of *S. aureus*

To identify if individual microbial community features correlated with *S. aureus* status, we fit linear mixed models for the association between *S. aureus* phenotype and microbial features (taxonomic and functional) for infants and mothers separately, as well as for the association between infant phenotypes and the related mother’s microbial features (Additional file 3). Different measurements of *S. aureus* phenotype were utilized in our models to fully capture carriage patterns (the “Methods” section), such as the “ever” acquisition of *S. aureus* over the study period, or positivity at a single time point by sequencing, culture, or either. We found that, of the covariates tested, daycare attendance in the preceding month produced the most significant changes in the infant microbiome (Fig. 3). More specifically, daycare attendance was associated with increases in the relative abundance of the pathogens *Moraxella catarrhalis* and *Haemophilus influenzae* (Fig. 3), which are also reported in the literature [44, 55, 56], and linked to the development of wheezing, asthma, and respiratory infection in infants [55, 57–60].

In the taxonomic subset of these linear models, abundances of infant *Acinetobacter* species, *Streptococcus parasanguinis*, *Streptococcus salivarius*, and *Veillonella* species were weakly positively associated with *S. aureus* acquisition by the next month’s sample (FDR *q* < 0.25, Fig. 3). These trends tended to be driven by acquisitions occurring at later time points; the presence of any of the four species was more frequently observed during acquisition events occurring after the first month (Fisher’s exact test, *p* = 0.0805). The nasal microbiome may thus play a larger role in *S. aureus* carriage patterns as infants get older, which is concordant with a diminishing maternal and increasing environmental influence over time. However, we were limited in our cohort in the number of acquisition events occurring after the first month, causing the significance of these associations—except for *S. parasanguinis*—to be somewhat dependent on the model specification (i.e., not always significant during robustness assessment, the “Methods” section, Additional file 1: Fig. S7). Although this finding is exploratory in nature, we recommend that future studies account for early and late acquisition events differently in order to ensure sufficient and balanced sample sizes.

Notably, there was no overlap in the predictors of *S. aureus* in infants and mothers, which is consistent with the significant taxonomic differences between the two. However, the maternal abundance of the commensal species *Dolosigranulum pigrum* was inversely associated with infant “ever” acquisition of *S. aureus*. A number of epidemiological studies have reported an inverse association between *D. pigrum* and *S. aureus* in adults [19, 36, 61, 62], as well as infants [63], although the potential mechanisms or causality of the relationship is unknown.

In our results, the two *Streptococcus* species positively associated with *S. aureus* gain in infants were both inversely associated with breastfeeding in the previous month (FDR *q* < 0.25). These findings are consistent with previous research on the infant nasopharyngeal microbiome at 6 weeks of age, which reported inverse relationships between breastfeeding and *Streptococcus* OTUs [64], as well as, interestingly, inverse associations for breastfeeding with *Veillonella* species and *S. aureus* [64]. Breastfeeding has been identified as a risk factor for *S. aureus* nasal carriage in infants [65] due to the presence of *S. aureus* on maternal skin and in breast milk [43] but was not associated with *S. aureus* carriage in this cohort [17]. This absence of an effect may occur due to the competing pressures of increased exposure to maternal *S. aureus* during breastfeeding with protective ecological changes to the infant microbiome promoted by breastfeeding.

We next assessed microbial functions (specifically gene families grouped by Enzyme Commission (EC) number) that were contributed by diverse members of the nasal community. To avoid gene families that recapitulated individual taxa, we removed functional features for which a single species contributed more than half the feature abundance in more than half of samples containing that feature. Among the infants, 81 ECs (of 443 total retained in infants) were associated with daycare, demonstrating the strong functional consequences of the corresponding structural rearrangement subsequent to daycare attendance. Fifty-six ECs (of 423 total in mothers) were associated with time in the mothers, indicating that the maternal nasal microbiome does change, if subtly, in the year after birth. Overall, few functions in the infant and maternal microbiomes were associated with infant *S. aureus* phenotypes: four to five ECs each (of 160 total) in the infants were associated with infant *S. aureus* positivity by sequencing, *S. aureus* gain, and persistent carriage. The only EC to be associated with a *S. aureus* phenotype in infants and mothers was EC 1.6.5.5, nominally annotated as a NADPH:quinone reductase, which was inversely associated with *S. aureus* positivity by sequencing in both populations; this feature is examined in detail below. There were 50 ECs (of 181 total) in the mothers significantly associated with infant “ever” acquisition of *S. aureus* over the study period, 44 of which were inverse associations. As was subsequently found to be linked with EC 1.6.5.5, further investigation showed that this signal was mainly driven by the maternal *D. pigrum* association with infant “ever” acquisition (Additional file 1: Fig. S2).

To further explore this and overall relationships between *S. aureus* and the nasal microbiome, we constructed random forest models to predict infant *S. aureus* phenotype using the taxonomic and functional composition of the infant and matched maternal microbiomes. Discriminative (e.g., random forest) rather than generative (e.g., linear) models can be used to identify a subset of features that most strongly differentiate populations with different phenotypes. In random forest models, prediction of infant *S. aureus* phenotypes using infant and maternal taxonomic profiles did not perform significantly better than chance. However, prediction accuracy improved slightly when using functional profiles; the prediction of *S. aureus* by sequencing using infant functions had an AUC of 0.748 with 95% CI [0.602, 0.895], while the prediction of infant “ever” acquisition of *S. aureus* using maternal functions had an AUC of 0.748 with 95% CI [0.538, 0.959].

To understand why prediction improved when using functions (compared to species), we pinpointed an uncharacterized protein that proved to be associated with *D. pigrum.* The functional feature most responsible for this improvement was gene family UniRef90 X5NU12, a small 42-amino acid sequence to which taxonomy was not assigned by our analysis. However, its abundance was moderately correlated with the relative abundance of numerous other genes contributed by *D. pigrum* (1358 genes with a Spearman’s rho 0.35–0.44, Additional file 1: Fig. S3). This gene family was of major interest because it was present in about half (96 of 208) of samples and made up the majority (92%) of all sequences assigned (likely incorrectly) to EC 1.6.5.5 (NADPH:quinone reductases). Thus, the gene family summarized as EC 1.6.5.5 was the primary random forest predictor of infant *S. aureus* status by sequencing (Additional file 1: Fig. S4) and was significantly inversely associated with *S. aureus* positivity by sequencing in both infants and mothers in linear modeling (Additional file 1: Fig. S5). This finding was not limited to our dataset—in healthy adults in the second phase Human Microbiome Project (HMP1-II) [66], UniRef90 X5NU12 was present in about half of anterior nares samples (131 of 251), and its relative abundance was significantly inversely correlated with *S. aureus* presence by sequencing (Wilcoxon *p* < 10^−5^ across 251 samples). A BLAST [67] search of reads mapping to this gene family revealed many significant alignments with 16S rRNA gene sequences nominally assigned to species as diverse as *Streptococcus*, *Staphylococcus*, *Lactobacillus*, *Cutibacterium*, and *Bifidobacterium*. Given these diverse results, closer inspection of the UniRef90 X5NU12 family suggests that it may be a misannotated fragment of the highly conserved 16S rRNA gene, with sequences matching these reads from previous skin and nasal studies potentially misannotated to a variety of closely related taxa (including *D. pigrum*). Thus, UniRef90 X5NU12 may be acting as a taxonomic marker for a closely related, uncharacterized species co-occurring or sharing genetic similarity with *D. pigrum* and competing with *S. aureus*. Meanwhile, many of the maternal ECs associated with infant *S. aureus* “ever” acquisition in the linear models contributed somewhat to the predictive power of the random forest for this variable, but none were highly dominant predictors like UniRef90 X5NU12 (Additional file 1: Fig. S6).

**Fig. 4:**
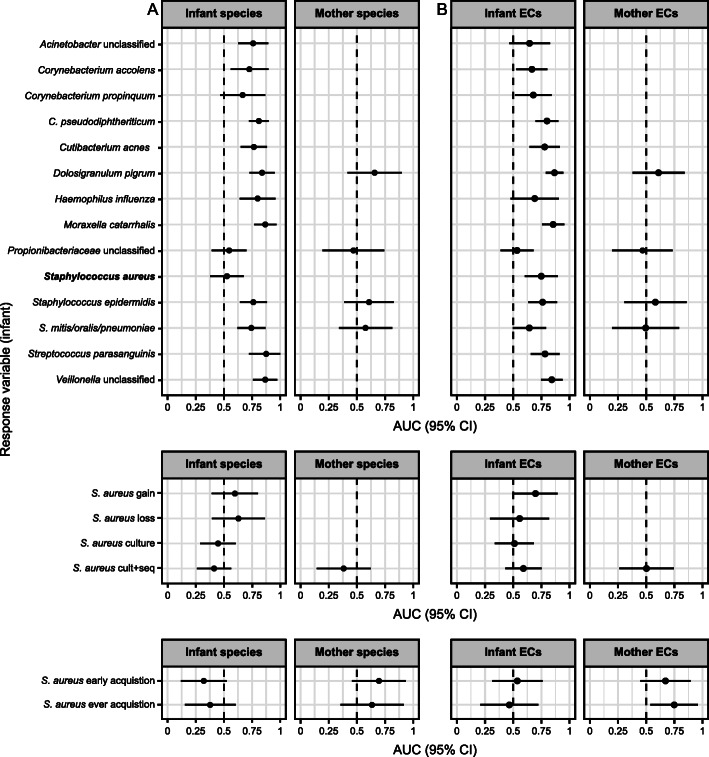
Ability of infant or maternal microbiome profiles to predict infant microbiome membership. Both infant and maternal **a** species-level relative abundance profiles and **b** functional profiles (ECs) were used as predictors in random forests to infer (1) the presence/absence of other individual species by sequencing, (2) subject-varying *S. aureus* variables, and (3) subject-fixed *S. aureus* culture phenotypes in infants. When predicting properties of a given species (presence/absence or any *S. aureus*-derived property), we **a** removed the species from the abundance table or **b** removed any ECs contributed to by the species and renormalized all samples to 100% prior to model fitting to avoid circularity. The infant microbiome exhibited reasonably strong within-ecosystem cohesion (i.e., predictability) but essentially none from the maternal to infant microbiome; conversely, only certain *S. aureus* carriage phenotypes were well predicted by this model

Random forest classifiers using infant species-level taxonomic and functional profiles were also able to successfully predict the presence/absence of a number of other taxa in the infant microbiome, including several potential pathogens and important commensals (Fig. 4). In total, 11 out of 13 of these models had AUCs that were significantly above 0.5 when using species as predictors (Fig. 4), and 9 out of 13 models had AUCs significantly above 0.5 when using functions (Fig. 4, bootstrap 95% CI > 0.5). Overall, the maternal microbiome was not predictive of the infant microbiome, with only one of the models achieving significance. This is consistent with our earlier analysis (i.e., Figure 2), as the random forest models are built using time-matched mother-infant samples from all time points, and mothers and their infants were significantly more similar at only one of 13 time points (and even then were still quite different from each other). Many species in the nasal microbiome appear to be interdependent, but in complex ways, thereby allowing moderate prediction by random forest models; however, *S. aureus* itself appears to be a more isolated member of the ecological community of the nose.

### Gene-level strain profiles reveal similarity between mother and infant *S. aureus*

**Fig. 5:**
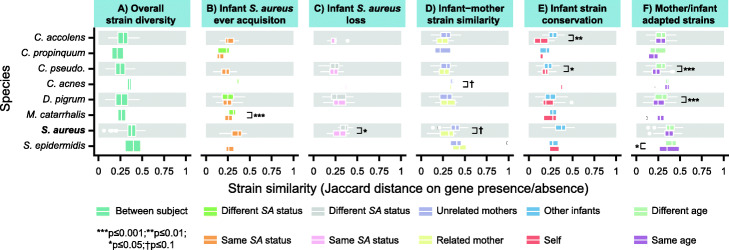
Gene content-based strain profiling for *S. aureus* and other common nasal species. To perform gene content-based strain profiling for each species, pairwise Jaccard distances were calculated between samples on accessory gene presence/absence and averaged over same-subject pairs. Species/distance metric combinations with fewer than three subject pairs formed from six fully unique subjects are not displayed (see Additional file 2 for sample sizes). Significance testing was performed with a one-sided Wilcoxon-Mann-Whitney test

To finally explore strain-level trends in *S. aureus* acquisition and carriage and put these in context relative to other common nasal species, we compared species-level gene content (i.e., strain-specific accessory genome carriage) across samples (Fig. 5). Given the limited sequencing depth available after human nucleotide depletion, strains were characterized based on the presence/absence of gene families in each species’ reference-based pan-genome [68, 69] rather than by calling individual nucleotide variants. Per species, we calculated the average Jaccard distance between accessory gene profiles in different subjects to quantify genetic diversity for that species in our population. We also compared the average Jaccard distance between sample pairs matched on a metadata variable of interest (e.g., mother-infant pair) to that between discordant sample pairs. For example, one test (Fig. 5b) considered whether infants were ever positive for *S. aureus* over the study period and compared the strain similarities (averaged over subject pairs) of concordant samples (i.e., both infants “ever” or “never” acquired *S. aureus*) compared to discordant samples (i.e., one infant “ever” acquired and one “never” acquired *S. aureus*).

Of the eight species with sufficient coverage to profile strains, the overall population mean genetic diversity/distance was highest in *S. epidermidis* and *S. aureus* and lowest in *Corynebacterium propinquum* and *Corynebacterium pseudodiphtheriticum* (Fig. 5a). For many of the species, gene content was more similar within an infant over time than compared to other infants (Fig. 5e), indicating stability of the strains over time (mirroring the behavior of the adult gut [66, 70, 71]), and this reached significance for *C. accolens* and *C. pseudodiphtheriticum*. Interestingly, individuals with the same infant/mother status (i.e., both mothers or both infants) had more similar gene content for *C. pseudodiphtheriticum*, *D. pigrum*, and *S. epidermidis* than individuals with differing infant/mother status (Fig. 5f), suggesting that these species may have strains adapted to microbiome maturity or be transmitted within age groups.

Infants carried strains of *S. aureus* that were somewhat more similar to those of their own mothers than to unrelated mothers (Fig. 5d) or other infants (*p* = 0.0520). This is consistent with the findings from the parent cohort study that infants often carried maternal strains [17], although we use different methods for defining similarity and, due to undersampling in our data and the high specificity of our accessory genome barcoding, infants rarely had identical strains to their mother. Interestingly, this similarity of the infant strain to the maternal strain was more pronounced in *S. aureus* compared to other species. Furthermore, infants who were positive for *S. aureus* by culture and were concordant on loss status (i.e., either both lost or did not lose *S. aureus*) had *S. aureus* with more similar gene content compared to individuals discordant on loss status (*p* = 0.0359) (Fig. 5c), suggesting some strains may be more ecologically stable, i.e., more difficult to lose once acquired. Strain-level variation in other species may also be related to *S. aureus* status; for instance, we found that infants with the same *S. aureus* “ever” acquisition status had very significantly more similar strains of *M. catarrhalis* (*p* < 10^−4^) (Fig. 5b).

## Discussion

In this study, we performed the first shotgun metagenomic analysis of the infant nasal microbiome, with a particular focus on *S. aureus* acquisition and retention. Using multiple measures of *S. aureus* status and maternally paired, longitudinal microbiome data, we sought to identify microbiome determinants temporally preceding changes in infant *S. aureus* status and to investigate the influence of developmental time and the maternal microbiome on the early infant microbiome. The infant nasal microbiome was highly variable over the first year and was primarily shaped by developmental and external factors (e.g., environmental exposures such as daycare). Using both linear and random forest models, as well as replication in healthy adults in the HMP1-II dataset, we found evidence of a gene family, possibly representative of an uncharacterized taxon, that was associated with *D. pigrum* and significantly anticorrelated *S. aureus*.

Generally, the taxonomic composition of our samples matched that seen in previous studies [42, 72] with *Moraxella*, *Streptococcus*, *Haemophilus*, *Staphylococcus*, *Corynebacterium*, and *Dolosigranulum* making up 71.78% of the total abundance of infant samples. In contrast to some previous studies, we identified *Cutibacterium* (previously *Propionibacterium*) as a major genus dominating the infant nasal microbiome in the first year of life. This difference is likely due to previous studies often sequencing hypervariable region 4 of the 16S ribosomal RNA (rRNA) gene, which has been shown to severely underestimate *Propionibacterium* abundance [73]. After *Dolosigranulum pigrum* (30.60%), *Cutibacterium acnes* had the second highest mean abundance among infants (18.44%). In a linear model, birth time points were significantly (*q* < 0.25) associated with *C. acnes* and *Propionibacteriaceae* unclassified (possibly *C. acnes* that could not be identified due to low sequencing read counts), which together made up 60.13% of the relative abundance of samples at birth. *D. pigrum* tended to dominate subsequently, and later (non-birth) time points were associated with significant elevations of *D. pigrum*, *C. pseudodiphtheriticum*, *M. catarrhalis*, and *S. tigurinus* (*q* < 0.25). Previous research has suggested that interaction with *C. acnes* can increase *S. aureus* virulence [74], helping *S. aureus* to invade the human host [74]. Similarly, the *C. acnes*-produced small molecule coproporphyrin III can promote *S. aureus* biofilm formation, potentially improving its survival in challenging environments [75]. In our study, just over half of infants gained *S. aureus* at or before month 1; in contrast to more complex *D. pigrum*-dominated microbiome compositions seen later in the first year, this low-diversity *C. acnes* dominated nasal microbiome at birth may fail to prevent or even promote *S. aureus* acquisition.

Our findings on infant nasal microbiome development further elucidate the results from the parent cohort study, which tracked *S. aureus* phenotypes using cultured isolates and defined strains by pulsed-field gel electrophoresis (PFGE) and *spa* typing (rather than sequencing) [17]. In the full cohort, it was found that 61.8% (*n* = 76) of the infants carried *S. aureus* at month 1, with the majority (72%, *n* = 52) carrying the same strain as the mother by PFGE/*spa* [17]. Our results suggest the mother is an important influence on the infant nasal microbiome at and before month 1, which could allow for the sharing of both commensal nasal species, as well as *S. aureus* and other pathogens. Our strain definition via sequencing also suggests that infant strains are often genomically similar, but not identical, to the most abundant strain in the mother. In the larger cohort, we found that early acquisition (by month 1) of *S. aureus* resulted in a longer length of carriage [17]. Here, in the early months, full metagenomic profiling made it clear that the nasal microbiome is undeveloped with low species alpha diversity and high *C. acnes*, which may allow *S. aureus* transmitted at this time to gain a strong foothold.

Although *S. aureus* transmitted by the mother may be present very early in life, the infant nasal microbiome and immune system undergo major changes throughout the first year of life that likely act to exclude *S. aureus*, resulting in declining rates of carriage in the year after birth. Consistent with studies of the gut microbiome, in which maternal influence after birth on vaginally delivered infants is quite strong for a short period of time, but then quickly fades [50, 52–54], we found that infants were not more similar to their own mother at later time points and that their taxonomic profiles overall rapidly diverged from the earliest time points. In contrast, environmental factors, especially daycare, strongly shaped the nasal microbiome in the first year, as it was associated with the acquisition of new species, and possible strain replacement for existing species.

Daycare attendance in particular was one of the largest effects in our study and the parent study, both on *S. aureus* specifically and the nasal microbiome generally. In the larger cohort, daycare was significantly negatively associated with persistent *S. aureus* carriage [17], and, in our subset, none of the three persistent carriers attended daycare at any point. Consistent with previous research [44, 55, 56], daycare attendance was marked by a large increase in the relative abundance of *M. catarrhalis* (Fig. 3). The likely inverse relationship between *M. catarrhalis* and *S. aureus* [76–78] and the acquisition of *M. catarrhalis* at daycare then explain why daycare attendance may reduce persistent carriage. Surprisingly, we also found that the three persistent carriers had higher *M. catarrhalis* relative abundance than other daycare non-attendees (Fig. 3). While this is not inherently contradictory, since none of these associations must be transitive per se, it may be explained by external confounding factors such as contact with siblings, since the presence of siblings is a risk factor for both *M. catarrhalis* [44, 79] and *S. aureus* colonization [65], or by random chance given the small sample size. However, there may also be a more interesting biological explanation, such as combinatorial interactions with additional species like *H. influenzae*, which is also acquired with daycare attendance, or strain differences in *M. catarrhalis*, as we did find a highly significant association of *M. catarrhalis* strain type with *S. aureus* “ever” acquisition (Fig. 5). Similar to *Corynebacterium* species, in which studies have reported inconsistent associations with *S. aureus* due to the possible complexity of these interactions [11], further in vitro work exploring the circumstances under which *M. catarrhalis* inhibits *S. aureus* would be valuable.

One of the other most striking observations in our data was the presence of a gene family, correlated with *D. pigrum* genomic content, that was strongly co-exclusionary with *S. aureus* carriage. Due to its similarity with 16S rRNA gene sequences, this gene family likely represents a species not closely homologous to current reference isolates that competes with *S. aureus*. Its correlation with *D. pigrum* in our dataset suggests that it may either occupy a related phylogenetic space or that these two species may act as co-colonizers. Furthermore, previous epidemiological studies have reported inverse associations between *D. pigrum* and *S. aureus* in adults [19, 36, 61, 62] and infants [63], and identified diverse biosynthetic gene clusters in *D. pigrum* believed to be involved in its in vitro inhibition of *S. aureus* [62]. In one study of hospitalized neonates, cases (those acquiring *S. aureus*) had lower *D. pigrum* relative abundance compared to matched controls 7 days prior to *S. aureus* acquisition [63], and a recent study found that 10 of 10 diverse *D. pigrum* isolates inhibited *S. aureus* growth in vitro [62]. The mechanisms through which *D. pigrum* inhibited *S. aureus* in this experiment were not fully clear, but one hypothesis explored by the authors was an enrichment of biosynthetic gene clusters (BGCs) in *D. pigrum*, with strains containing both a lanthipeptide BGC and a bacteriocin inducing the greatest inhibition [62]. Our finding of the inverse association between maternal *D. pigrum* and infant “ever” acquisition of *S. aureus*, however, is the first to suggest that *D. pigrum* is one pathway through which the maternal microbiome influences infant *S. aureus* carriage. From our results alone, it is not clear whether *D. pigrum* prevents the acquisition of maternal *S. aureus* that could be passed to the infant, or whether maternal *D. pigrum* is passed to the infant where it is protective against *S. aureus* acquisition, and in either case, whether *D. pigrum* itself or an uncharacterized relative is responsible for the additional sequences co-exclusionary with *S. aureus*.

Due to the cost of metagenomic sequencing and the complexity of *S. aureus* carriage, our study has some limitations. Firstly, our data is collected monthly, which may be too coarse a temporal resolution for understanding the full relationship between *S. aureus* status and the rapidly developing infant microbiome. Secondarily, we are limited by the size of our sample, and this is ultimately an exploratory analysis whose findings should be tested in larger cohorts. In particular, this creates challenges if early and late *S. aureus* acquisition events indeed are driven by different influences (e.g., maternal *S. aureus* carriage vs. the establishing infant nasal microbiome), as this creates a mixed signal with an already limited sample size and is worthwhile to consider for future study design. Additionally, using metagenomics for skin habitats is challenging due to the high degree of human contamination, and we were not able to perform nucleotide-level strain analysis given the relatively low sample read depths. Lastly, *S. aureus* carriage is a complex phenomenon, with ecological and microbiological variation undoubtedly contributed by unmeasured factors (e.g., immune development, contact with other caregivers and relatives, diet), and therefore, microbiome contributions need to be large to be detectable. Particularly, during highly dynamic infant colonization, a wide range of environmental influences (in addition to direct maternal effects) are likely to influence microbiome composition. These effects are further confounded since the mother’s nasal (and other) microbiome composition may in turn be influenced by some of these same factors due to shared genetics, cohabitation, lifestyle factors, and/or transmission of strains with other children, all of which have been demonstrated for the gut microbiome [52, 80–86]. Through an integrative microbiome epidemiology study design, we have provided new insights into the development of the infant nasal microbiome and its role in *S. aureus* carriage that suggest further targets of investigation for eventual ecological therapies to control *S. aureus* carriage.

## Conclusions

In this study, we characterized the development of the infant nasal microbiome, which was highly variable, weakly influenced by the maternal nasal microbiome composition, and strongly shaped by daycare attendance. Mothers thus represented a sporadic early source for *S. aureus* transmission to the naive infant microbiome, but microbiome determinants became more important later on. We gained a better understanding of the role of the microbiome in *S. aureus* carriage through the identification of a specific protein family that was highly predictive of infant *S. aureus* status, significantly anticorrelated with *S. aureus* positivity in both infants and mothers, and which ecologically interacts with the commensal species *D. pigrum*. We determined that this (misannotated) protein family was a non-protein-coding sequence acting as a phylogenetic marker of a likely novel bacterial species. An inverse relationship between *D. pigrum* and *S. aureus* has been a main result of multiple prior 16S rRNA amplicon studies; however, using metagenomic sequencing, we were able to differentiate this novel species from *D. pigrum* (which it often co-occurs with) and have shown that it is more predictive of *S. aureus* presence than is *D. pigrum* itself (with our result thus representing a likely mechanistic “driver” rather than previously identified “passengers”). Furthermore, this novel taxon was sufficiently prevalent in adults and infants to drive widespread patterns of *S. aureus* carriage. These findings were not limited to our cohort: a similar prevalence and a strong anti-correlation with *S. aureus* were also found for this novel clade in geographically distinct adults from the Human Microbiome Project. Our study provides an improved understanding of how the infant nasal microbiome develops in early life, and how it can act to promote or exclude *S. aureus* colonization.

## Methods

### Cohort and experimental design

Subjects consisted of 36 mother-infant pairs recruited through Sheba Medical Center as part of a larger cohort study on *S. aureus* [17], as well as *S. aureus*-negative mothers enrolled specifically for this study. More specifically, subjects were recruited from among pregnant women who attended the Sheba Medical Center obstetrics monitoring unit between 2013 and 2016, were at least 34 weeks pregnant, visited the monitoring unit during screening hours (held for three hours per week), and provided consent to participate. Exclusion criteria included delivery prior to 34 weeks of pregnancy and failure to provide consent to participate. Nasal swabs and subject covariates were collected from mothers and infants within 48 h of birth and then monthly for the first year after birth. At each time point, two swabs (one for *S. aureus* culture testing and one for DNA extraction and sequencing) were collected from both nostrils. The covariates included infant sex, maternal age, delivery mode, daycare attendance in the past month, antibiotic use in the past month, and breastfeeding in the past month (Additional file 4). Culture testing for *S. aureus* (detailed below) was performed for all non-missing samples (*n* = 856), and 33.2% of samples (*n* = 284) were sequenced with shotgun metagenomic sequencing (also below, and see Fig. 1). As much as possible, shotgun-sequenced samples were selected to target birth and the first month for all subjects, *S. aureus* gain and loss events in infants, and extended periods of *S. aureus* carriage or non-carriage in infants.

### *S. aureus* culture testing

Nasal screening was performed using a cotton-tipped swab placed in Amies transport media (Copan innovation, Brescia, Italy). Swabs were streaked on CHROMagar *S. aureus* plates (HiLabs, Rehovot, Israel) within 24 h and incubated for 24–48 h at 35 °C. Catalase and Staphylase (PASTOREX® STAPH-PLUS, BioRad, Marnes-la-Coquette, France) were performed on suspected colonies to conclusively identify them as *S. aureus*.

### DNA extraction and sequencing

DNA extraction was performed with the QIAGEN DNeasy PowerLyzer PowerSoil Kit with a bead-beating protocol. After extraction, DNA concentration was measured by NanoDrop and frozen at − 20 °C until being shipped to the USA for sequencing. Prior to starting the project, the DNA extraction method was validated by performing extractions on double-distilled water (DDW) samples and confirming that no DNA concentration was measured by NanoDrop and that the displayed band was a true band and not due to contamination. Negative controls were not additionally sequenced since specimen DNA concentrations were measured after extraction to be reliably high and differentiated from potential background contaminants (due to the otherwise substantial added cost).

Whole-genome fragment libraries were prepared as follows at the Broad Institute [87]. Metagenomic DNA samples were quantified by Quant-iT PicoGreen dsDNA Assay (Life Technologies) and normalized to a concentration of 50 pg/μL. Illumina sequencing libraries were prepared from 100 to 250 pg of DNA using the Nextera XT DNA Library Preparation kit (Illumina) according to the manufacturer’s recommended protocol, with reaction volumes scaled accordingly. Prior to sequencing, libraries were pooled by collecting equal volumes (200 nl) of each library from batches of 96 samples. Insert sizes and concentrations for each pooled library were determined using an Agilent Bioanalyzer DNA 1000 kit (Agilent Technologies). Libraries were sequenced on HiSeq 2 × 101 to yield ~ 10 million paired-end reads. Post-sequencing de-multiplexing and generation of BAM and Fastq files were generated using the Picard suite.

### *S. aureus* phenotype definitions

The *S. aureus* phenotype of a sample was defined using multiple measures to capture different dimensions of *S. aureus* carriage (Additional file 5). Subject-fixed *S. aureus* phenotypes were defined strictly by the *S. aureus* culture data and included *S. aureus* early acquisition, late acquisition, “ever” acquisition, persistent carriage, the percent of positive time points, and the time point of first *S. aureus* acquisition. *S. aureus* early acquisition applied only to infants, and was defined as first acquisition of *S. aureus* by or at the first month. *S. aureus* late acquisition also applied only to infants and was defined as first acquisition of *S. aureus* after month 1. If culture results for birth were missing, early and late acquisition phenotypes were calculated using month 1 data only. *S. aureus* “ever” acquisition for both mothers and infants was defined as ever being positive for *S. aureus* by culture over the study period. To match definitions in the larger cohort study [17], infant persistent carriage was defined as being positive for at least two thirds of non-missing time points and being positive for at least half of non-missing time points between months 6 and 12. For mothers, persistent carriage was defined as being positive for at least two thirds of non-missing time points. For infants and mothers, the percent of positive time points was defined as the number of positive time points divided by the number of non-missing time points for the subject over the study period. For infants, the time point of first *S. aureus* acquisition was defined as the first time point for which the subject tested positive for *S. aureus* or NA for subjects who never tested positive.

Subject-varying *S. aureus* phenotypes included positivity by culture or sequencing or either, gain of *S. aureus* by the next month among culture-negative samples, and loss of *S. aureus* by the next month among culture-positive samples. Positivity by sequencing was defined as having a non-zero *S. aureus* relative abundance. Positivity by either was defined as being positive by culture or sequencing or both. Due to the false-negative rate from culture, the culture data was smoothed according to the following procedure: (1) negative or missing data points that were preceded and followed by positive data points were treated as positive, and (2) missing data points that were preceded and followed by negative data points were treated as negative. Using the smoothed culture dataset, *S. aureus* gain by the next month was defined as changing from negative by culture at month *t* to positive by culture at month *t* + 1. *S. aureus* loss by the next month was defined as changing from positive by culture at month *t* to negative by culture at both months *t* + 1 and *t* + 2. Figure 1 shows the raw, unsmoothed data.

### Data processing and quality control

Three sequenced samples failed our sample tracking quality control and were excluded from further analysis. Samples were retained in the analysis if they contained more than 5 × 10^4^ sequenced read pairs (mean 9.47 × 10^5^ read pairs per sample) and were not taxonomically 100% unclassified (detailed below). This cut-point was selected a priori based on approximately 1× coverage of an *Escherichia coli*-equivalent genome size. Of the 284 sequenced samples, a total of 208 samples (144 from infants and 64 from mothers) were retained for further analyses.

Taxonomic and functional profiles were generated using the bioBakery meta’omics workflow v0.9.0 [88]. Briefly, reads mapping to the human genome were first filtered out using KneadData v0.7.0 with default parameters. Taxonomic profiles of shotgun metagenomes were generated using MetaPhlAn2 v2.6.0, and functional profiling was performed by HUMAnN2 v0.11.0. In taxonomic profiles, relative abundances were given at the species level or, if unidentified, at the genus level or family level. Phage abundance was originally included in the taxonomic profiles, but due to the lack of any significant associations (*q* < 0.25) in linear models, phage abundance was normalized out and subsequent analyses focused purely on non-viral taxa. In functional profiles, per-sample gene abundances were grouped by Enzyme Commission (EC) number using the HUMAnN2 utility script.

Due to the presence of quality-controlled DNA that did not map, we treated the unmapped sample mass like a microbial feature (i.e., rescaled the taxonomic profiles by the percent mapped reads and included a feature for the percent unmapped reads) and assessed the robustness of our results using these new profiles. The percent of unmapped reads was strongly correlated with human contamination (Spearman rho = 0.69, *p* < 10^−15^ across all 208 filtered samples) and did not change our main findings from linear models (Fig. 3, Additional file 1: Fig. S7), other than reducing power (due to noise introduction and re-removal) and causing some weaker associations to drop out. We concluded that much of this unmapped sample mass represented mainly cryptic human contamination and that our initial approach of focusing purely on mapped reads and adjusting linear models for the proportion of human contamination was robust.

### Statistical methods for assessing infant developmental trends

Alpha and beta diversities were calculated using the Simpson index and Bray-Curtis dissimilarity, respectively, in the *vegan* R package v2.5.4. For mothers, beta diversity was calculated using average per-subject taxonomic composition (due to limited sample sizes). In comparisons between infants with different *S. aureus* statuses, *S. aureus* relative abundance was normalized out prior to the calculation of diversity metrics. Significance testing was performed using a one-sided Wilcoxon-Mann-Whitney test. Any time points with fewer than five data points were not included in this analysis (see Additional file 2 for sample sizes). PERMANOVA was performed using the *adonis* function within the *vegan* R package. We tested the following variables using blocked PERMANOVA [87]: *S. aureus* early acquisition, late acquisition, “ever” acquisition, and persistent carriage. Within-subject permutations were used to test the effect of time (months). We used repeated cross-sectional PERMANOVAs to test: *S. aureus* positivity by culture, sequencing or either, and infant vs. mother status.

### Feature filtering

Prior to the creation of linear and random forest models, we filtered taxonomic and functional features to retain analyzable subsets of these features passing quality control. All filtering was applied separately to infant and mother samples due to the differences in microbiome composition, and all cut-points for filtering were selected a priori based on best practices from similar studies. First, all feature abundances below 0.1% (taxonomy) or 0.05% (function) abundance in more than 95% of samples were aggregated to a single “other” category to avoid testing features that were too uncommon to possibly detect an effect. Next, unless otherwise noted, microbiome features trivially related to the outcome variable of interest were dropped and the remaining per-sample abundance was renormalized to 100%. For example, *S. aureus* abundance was removed from infant taxonomic profiles in this way prior to the prediction of infant *S. aureus*-related variables in the random forest models. ECs were considered related to the outcome of interest if they contained a stratification for the outcome species in any infant or mother sample. Lastly, we applied a filter to remove functions that were primarily contributed by a single species to avoid re-analyzing functions that essentially recapitulated the genomic abundances of a single dominant carrying species. Specifically, if a single species (excluding unclassified) contributed more than 50% of a functional feature’s abundance in more than 50% of samples containing that feature, it was aggregated to an “other” category as above. Functions contributed by unclassified species were identified through the HUMAnN2 translated search (but not MetaPhlAn2) and were retained because they were not previously captured in our taxonomic analysis.

### Linear models

Linear mixed effects models to identify significant associations between microbiome features and covariates were created in MaAsLin2 v0.2.2. Briefly, log-transformed taxonomic and functional feature abundances were modeled as outcomes of a function of covariates, including a random effect for the subject and fixed effects for the *S. aureus* phenotype, time point (months), proportion of human contamination (defined as one minus the proportion of reads after vs. before human depletion), and total read count. We created models combining covariates and outcome features for (1) infant features and infant metadata, (2) mother features and infant metadata, and (3) mother features and mother metadata. Therefore, each class of model was defined by the choice of microbiome feature type (taxonomic or functional), *S. aureus* phenotype, and the three feature/metadata configurations listed above (Fig. 3). Within each unique combination of these levels, FDR correction was applied per variable using Benjamini-Hochberg, and the results with *q* < 0.25 are displayed (Fig. 3).

Models were fit for both subject-fixed and subject-varying *S. aureus* phenotypes. For subject-fixed phenotypes, such as “ever” acquisition of *S. aureus*, all samples were given the subjects’ phenotype value and included in the analysis. For subject-varying *S. aureus* phenotypes, such as sample positivity by sequencing, each sample with a non-missing value for the phenotype variable was included in the analysis. For *S. aureus* gain and loss, the analysis was subset to samples that were negative or positive for *S. aureus* by culture, respectively.

Other recorded exposures or environmental factors, such as antibiotic usage or delivery mode, were also evaluated as covariates potentially contributing to the relationship between microbiome features and the *S. aureus* phenotype. For mothers and infants separately, we included covariates in the model when (1) the covariate was significantly (*q* < 0.25) associated with at least one species in a univariate prescreen, and (2) the covariate was at present in at least 10% of samples (including those that were missing covariate data). Furthermore, models for maternal microbiome features were not adjusted for variables considered primarily related to infants, such as breastfeeding and daycare. These criteria were decided upon a priori. The rationale for (2) was that these covariates were not able to be tested in our dataset because they were too rare to be individually significant. Based on these criteria, models for infant microbiome features were also adjusted for breastfeeding and daycare in the preceding month, while models for maternal microbiome features were not adjusted for any additional covariates. The displayed results (Fig. 3) for non-*S. aureus* metadata are from a model containing all covariates except a *S. aureus* phenotype.

### Random forest models

Random forest classifiers were created using the *randomForest* R package v4.6.14. Across models, the predictors consisted of filtered infant and maternal taxonomic and functional features. The binary response variable was defined as the infant *S. aureus* phenotype or the presence/absence of a species in the infant nasal microbiome by sequencing (the species tested were those that passed the prevalence/abundance filter). We did not study response variables for the mothers due to the lower sample size. As described above, features were normalized out of the predictor dataset if they were related to the outcome variable in models constructed with infant predictors/infant outcomes.

The *randomForest* function was run with default parameters, with the exception of downsampling to the size of the smaller class to handle the unbalancedness in the data. To conservatively address subject-level effects, cross-validation was performed per subject: iterating through each subject, a test dataset containing all samples from the same subject was created, the model was trained on data from all other subjects, and then the outcome for the excluded individual was predicted. To find 95% confidence intervals, the standard error was calculated through per-subject bootstrapping, with 1000 bootstrap samples used. Random forest models were only run if they had at least 20 samples in each class (i.e., a sufficient size for meaningful cross-validation) (see Additional file 6 for sample sizes).

### Strain-level profiling

Given the limited sequencing depth available after human nucleotide depletion, strain profiles were generated using PanPhlAn v1.2.2, which characterizes strains based on the presence/absence across gene families from across each species’ reference-based pan-genome [68].

To create Fig. 5, per-species gene presence/absence matrices were first filtered to remove genes present in less than 5% of samples for which PanPhlAn was run. The *vegdist* function in the *vegan* R package v2.5.4 was used to calculate pairwise Jaccard distances between samples based on a metadata variable of interest. For each comparison in Fig. 5, pairwise distances were averaged over same subject pairs to avoid undue influence by subjects with more sequenced time points. Comparisons were displayed if they had data from at least three unique pairs consisting of six unique subjects. This was to avoid bias that could occur if many pairs were created with the same subject and this subject happened to have unusual data. Significance testing was performed using a one-sided Wilcoxon-Mann-Whitney test. Due to low sample read depths, underdetection of genes is likely; however, the underdetection should not be differential with respect to the metadata variable of interest, and therefore, the significance testing is unbiased.

## Supplementary Information

**Additional file 1:** All supplementary figures.

**Additional file 2:** **Table S1**. Sample sizes for boxplots.

**Additional file 3:** **Table S2**. Full linear model results for Fig. 3.

**Additional file 4:** Summary of metadata for study population and samples collected for *S. aureus* microbiome profiling.

**Additional file 5: Table S4**. Summary of *S. aureus* variables for study population and samples collected for *S. aureus* microbiome profiling.

**Additional file 6: Table S5**. Sample sizes for random forest models.

**Additional file 7: Table S6**. Subject metadata.

**Additional file 8: Table S7**. MetaPhlAn2 taxonomic profiles.

**Additional file 9: Table S8**. HUMAnN2 functional profiles.

**Additional file 10:** Review history.

## Data Availability

The metagenomic sequences generated and analyzed during the current study are available in the NCBI Sequence Read Archive (SRA) repository as BioProject PRJNA610982 [89]. The subject metadata and the processed taxonomic and functional datasets generated and analyzed during the current study are included in this published article (Additional files 7, 8, and 9, respectively). All software used in this study is free and open source.
