# Supplementary material for: Determinants of Staphylococcus aureus carriage in the developing infant nasal microbiome
Source: Genome Biol. 2020 Dec 11;21:301. doi: 10.1186/s13059-020-02209-7 (PMC7731505; doi:10.1186/s13059-020-02209-7)
Supplement: Supplementary file 1 — Additional file 1: All supplementary figures. [file 13059_2020_2209_MOESM1_ESM.docx]

**Figure S1: *S. aureus* test concordance. (a)** Detection of *S. aureus* by culture and sequencing showed strong, but not complete, concordance, due to high rates of false negatives. **(b)** Sequencing false negatives (i.e., samples positive for *S. aureus* by culture, but with zero *S. aureus* relative abundance by sequencing) tended to have overall lower sample read depth compared to sequencing true negatives.

**Figure S2: PCoA plots of maternal taxonomic and functional features show associations with infant *S. aureus* “ever” acquisition.** Principal coordinate analysis (PCoA) plots were created using Bray-Curtis dissimilarity. Subjects with at least one sample with more than 25% *D. pigrum* relative abundance are displayed with a unique color. **(a)** As identified in the linear modeling results (**Fig. 3**), maternal relative abundance of *D. pigrum* was inversely associated with their infant “ever” acquiring *S. aureus* over the study period. **(b)** This association created a pronounced signal in the functional data (as also seen in **Fig. 3**).

**Figure S3: Spearman correlations of UniRef90 X5NU12 with the species-stratified abundances of other UniRef90s.** The histogram shows all correlations greater than 0.35, and is colored to show the species of origin. *D. pigrum* contributed the large majority of the abundance of species-stratified UniRef90s moderately positively correlated with UniRef90 X5NU12.

**Figure S4: Variable importance plot for the prediction of infant *S. aureus* status by sequencing using infant ECs.** In random forest models, EC 1.6.5.5 (NADPH:quinone reductase) in the infant nasal microbiome was the dominant predictor of infant *S. aureus* status by sequencing.

#####

**Figure S5: Association of EC 1.6.5.5 (NADPH:quinone reductase) with infant and mother *S. aureus* status by sequencing.** EC 1.6.5.5 was significantly inversely associated with *S. aureus* positivity by sequencing in both infants and mothers.

**Figure S6: Variable importance plot for the prediction of infant “ever” acquisition of *S. aureus* using maternal ECs.** In random forest models, a number of maternal ECs were important for the prediction of infant “ever” acquisition, but none were as dominant as EC 1.6.5.5 in the prediction of infant *S. aureus* status by sequencing (**Fig. S4**).

**Figure S7: Significant associations between nasal microbiome taxonomy and subject phenotypes in a sensitivity analysis of the unmapped sample mass.** The unmapped sample mass was treated like a taxonomic feature (i.e., taxonomic profiles were rescaled by the percent mapped reads, and the percent unmapped reads was included as a feature), and linear models were rerun. Significant associations (q<0.25) between individual taxonomic features and phenotypic covariates using a MaAsLin multivariable linear model for the rescaled profiles are shown. An annotation of “+” indicates that the association was not seen in the original (**Fig. 3**) linear models, and an annotation of “-” indicates that the association was only seen in the original (**Fig. 3**) linear models. Overall, the percent unmapped sample mass was mostly representative of human contamination. As a result, model power was reduced by inducing a relationship between taxonomic composition and human contamination, and then also subsequently re-adjusting for human contamination as a linear model covariate (**Methods**), causing weaker associations to drop over the threshold of significance.
