# Supplementary material for: Determinants of Staphylococcus aureus carriage in the developing infant nasal microbiome
Source: Genome Biol. 2020 Dec 11;21:301. doi: 10.1186/s13059-020-02209-7 (PMC7731505; doi:10.1186/s13059-020-02209-7)
Supplement: Supplementary file 10 — Additional file 10: Review history. [file 13059_2020_2209_MOESM10_ESM.docx]

**First round of review**

**Reviewer #1**

**Comments to author:**

In the manuscript "Determinants of Staphylococcus aureus carriage in the developing infant nasal microbiome", the authors presented the first shotgun metagenomic study of infant nasal microbiome, paired with maternal features, culture-based evidence of SA carriage and longitudinal sampling. Although some of the findings in the study are inconclusive (partly due to the nature of skin mWGS), the study provides insights to microbial and non-microbial features potentially responsible for the acquisition and stability of the nasal microbiome in general, and SA population in particular. Below are listed several points that might help to validate the robustness of the findings. 

1. Findings of the study (more specifically the linear modeling and random forest analyses) rely heavily on data stratification. Specifically, the linear model and the random forest model used at least 4 somewhat subjective (even if they are chosen to match a previous study) thresholds: 
1) first acquisition of SA by or at [the first month]
2) SA positive for at least [two-thirds] of all time points, and at least [half] of the time points between month [6-12].
Note that these factors could compound the limitaion of sample size.
Would the authors consider perturbing these parameters, so as to assess the robustness of the findings?

2. Taxonomic features used in the study were extracted using MetaPhlAn. Although the tool is accurate with regard to the identified taxa, the relative abundance estimates did not account for unknown taxa and therefore might not be comparable across samples. This is especially concerning as infant nasal mWGS has not been previously studied. Can the authors assess the consistency of these feature vectors, to ones that took into account unidentified taxa (e.g. using the new MAG catalog PMID: 30661755 that some of the authors have participated in generating)?

3. It is unclear to me how "strain" is defined in the study. It looks like a strain is defined by the significance of similarity (367-369, infants carry maternal strain because the gene content is significantly more similar than an unrelated mother), instead of the effect size of similarity (which is a more common definition). It is also questionable if similarity in gene content presence/absence (which is unlikely clock-like given the time scale) could infer transmission (365-366).

4. The authors suggested that infant strain gene contents are similar to their mothers', while 1) age-group adaptation could serve as a confounding factor, 2) infant microbiome is dynamic, 3) daycare has a strong influence on overall microbiome composition. In this case, does the gene content similarity between mother-infant pairs decrease overtime/correlate with daycare attendance?

5. (132-133) "..time points immediately preceding and following changes in S. aureus carriage status (were selected for sequencing)" does not seem to hold (for example, Row 4 in Fig. 1 - infant SA/early).

6. Panel labels (a/b/etc.) were not included for Fig. 3 and Fig. 4.

**Reviewer #2**

**Comments to author:**

Accorsi et al analyzed the Staphylococcus aureus carriage in the developing infant nasal microbiome, and studied the potential determinants of the carriage. The authors included 208 datasets and information derived from cultivation. The manuscript is well elaborated and is scientifically sound. The topic is interesting for a broad readership.

The study has some minor shortcomings (see below), including the selection procedure of the samples ("time points immediately preceding and following changes in S. aureus carriage status as determined by culture test in infants") and the high drop-out rate of samples due to low quality sequencing results, resulting in some time points with only a few datasets available (e.g. for month 9, infant). This restriction impairs the overall consistency, but probably does not affect the results.

As the authors state, this is one of the first reports to describe the development of the nasal microbiome of infants via metagenomics sequencing. Considering this, I would propose to include general information on the taxonomic and functional composition of the microbiome, and focus to Staphylococcus later in the manuscript. 

Also with respect to the viral/phage load, the sample information could be exploited more. As Staphylococcus phages are largely abundant in nasal samples, I consider they might also influence the nasal microbial community substantially. Viral/phage information could be obtained from the metagenomics dataset without large effort, and the derived results could be included in the manuscript.

My comments:

Line 43 and elsewhere: it is true, 284 samples were sequenced, but only 208 samples were retained for further analyses. Please correct throughout, as mentioning the higher number is somewhat misleading.
Page numbers are missing in pages before line 166.
Spaces are missing before the brackets for the references throughout the manuscript.
Line 86: correct: "Esp that that inhibits"
Line 94 to 108: This chapter could be condensed, as some of the information is partially redundant.
Line 147: can you explain the large difference between mean and median?
Line 150-151: this explanation is very vague; you would expect the sequencing approach to be more sensitive and to also detect non-cultivable ones; maybe extend the explanations a bit.
Fig. 1: I propose to split the figure into two panels, one for the culture results, one (next to it) for the sequencing result; the grey rims of the boxes are very difficult to identify. Extend the Figure legend and mention explicitly that the carriage (title of the legend) was analyzed via cultivation. Explain the colors also in the legend. Number the infant-mother pairs (each row).
Fig. 2: I propose to show the same resolution of data also for mothers (for those time points that are possible) in Fig. 2A, in order to visualize the variability also in this dataset. In Fig. 2B1: please explain the line at timepoint 0. In Fig. 2B3: highlighting of the significance now refers also to Fig. 1b1, so maybe indicate not associated to the sampling months but differently.
Line 205: Check sentence ("if"missing?)
Line 228: "This is interesting" - please rephrase
Line 230 ff: This part is largely discussion and could be moved to the discussion section.
Fig. 3: a) and b) should be included in the figure.
Line 292: please specify: not assigned to any level of taxonomy?
Line 319/20: significantly should only be used in case of a statistically significant finding; so maybe rephrase
Fig. 4: a) and b) should be included in the figure.
Line 431: "per se" should be in italics
Line 443: similarity with 16S rRNA gene sequences
Line 444: completely uncharacterized? Not even on domain level?
Line 470: this is a very important point: only mothers were included in the study, but also the other parent and siblings could contribute substantially; also skin contact during breast-feeding… consider to extend this part 
Line 499 ff: please identify inclusion and exclusion criteria for the cohort
Line 520: please explain how the DNA was extracted; were controls included?
Line 572: title should be changed, as it should refer to a methodological approach
Line 589 and elsewhere: the dataset was frequently filtered using certain cut-offs. I would be interested to know whether other cut-offs have been tested, or this was based on previous experience/ reference information? Please clarify.

**Authors Response**

**Point-by-point responses to the reviewers’ comments:**

# Reviewer #1:

*In the manuscript "Determinants of Staphylococcus aureus carriage in the developing infant nasal microbiome", the authors presented the first shotgun metagenomic study of infant nasal microbiome, paired with maternal features, culture-based evidence of SA carriage and longitudinal sampling. Although some of the findings in the study are inconclusive (partly due to the nature of skin mWGS), the study provides insights to microbial and non-microbial features potentially responsible for the acquisition and stability of the nasal microbiome in general, and SA population in particular. Below are listed several points that might help to validate the robustness of the findings.*

We very much appreciate the reviewer’s time spent on the manuscript and thoughtful comments to improve the work, particularly their favorable overview. As the reviewer pointed out, our study aims to provide a step forward in understanding of the infant nasal microbiome and *S. aureus* acquisition by including maternal microbiome sequencing, culture-based assessment of *S. aureus*, and longitudinal sampling.

*1. Findings of the study (more specifically the linear modeling and random forest analyses) rely heavily on data stratification. Specifically, the linear model and the random forest model used at least 4 somewhat subjective (even if they are chosen to match a previous study) thresholds:*

*1) first acquisition of SA by or at [the first month]*

*2) SA positive for at least [two-thirds] of all time points, and at least [half] of the time points between month [6-12].*

*Note that these factors could compound the limitation of sample size.*

*Would the authors consider perturbing these parameters, so as to assess the robustness of the findings?*

We agree these are the main parameters of our experimental design, initially created primarily by our clinical coauthor Dr. Gili Regev-Yochay. We’ve now both included a newly expanded perturbation analysis of the parameters, and focused primarily on the subset of phenotypes without parameters. As Reviewer #1 pointed out, persistent carriage (their definition #2) was defined *a priori* to match definitions used in the larger cohort study (Reiss-Mandel *et al.* 2019). The main findings of this study, which we focused on in the text, were exclusively for the subset of *S. aureus* phenotypes with specific definitions that did not depend on a cut-point. These included gain or loss of *S. aureus* by the next time point, ever-carriage of *S. aureus* over the study period, and *S. aureus* positivity by sequencing at a specific time point. Therefore, our main results presented in the manuscript do not depend on the choice of the cut points used in the *S. aureus* phenotype definitions #1 and #2 above.

In our original analysis, we included a continuous version of definition #2 above called “percent positive time points”. This was a subject-fixed *S. aureus* phenotype calculated using the *S. aureus* culture data, and for infants and mothers it was defined as the number of positive time points divided by the number of non-missing time points for the subject over the study period (thus not parameter-dependent). In the linear models, this variable was not associated with any taxonomic features, and thus is not shown in **Fig. 3A**. In **Fig. 3B**, the percent of positive time points (labeled as “% SA positive time points” along the x-axis) in infants was positively associated with the abundance of EC 4.2.1.24 (porphobilinogen synthase) in mothers with a borderline q-value of 0.189. Due to the lack of substantial results, this variable was also not included in the random forest modeling. **Fig. 3** and its caption are quoted here for reference:

##### “**Figure 3: Significant associations between nasal microbiome taxonomic and functional composition and subject phenotypes in feature-wise testing.** Significant associations (q<0.25) between individual taxonomic and functional features and phenotypic covariates using a MaAsLin multivariable linear model. **(a)** Of note, first attendance at daycare (during the preceding month) was an extremely strong determinant of initial microbiome colonization by several taxa (*M. catarrhalis, H. influenzae*), and the nasal microbiome exhibited a much weaker time-dependence than does the gut during infant development (i.e. few taxa were consistently temporally variable between months 0 and 12). The presence of a number of oral-associated species (i.e. *Streptococcus* and *Veillonella* species) was a mild correlate of *S. aureus* gain. *S. aureus* relative abundance was included as a positive control. **(b)** After applying a species-dominance filter, we found the association of many gene families (grouped by Enzyme Commission number, ECs) in infants with daycare attendance, while many functions in mothers were associated with time and infant “ever” acquisition of *S. aureus.*”

In response to the reviewer’s feedback, we have also repeated the linear modeling using a continuous version of definition #1 above. This was a subject-fixed *S. aureus* phenotype calculated using the *S. aureus* culture data, and defined as the first time point for which an infant tested positive (or NA for those subjects who never tested positive). We found that this variable was not significantly associated with any taxonomic or functional features in infants (consistent with the results for *S. aureus* early acquisition). Again, due to the lack of results, this variable was not included in the random forest modeling. We have updated **Supplementary Table 2**, **4**, and **5** to include information on this variable and the modeling results for this variable.

Additionally, we updated the “*S. aureus* phenotype definitions” section of **Methods** as follows to include a description of the variable:

###### “The *S. aureus* phenotype of a sample was defined using multiple measures to capture different dimensions of *S. aureus* carriage. Subject-fixed *S. aureus* phenotypes were defined strictly by the *S. aureus* culture data, and included *S. aureus* early acquisition, late acquisition, “ever” acquisition, persistent carriage, the percent of positive time points, and the time point of first *S. aureus* acquisition...For infants, the time point of first *S. aureus* acquisition was defined as the first time point for which the subject tested positive for *S. aureus* or NA for subjects who never tested positive.”

*2. Taxonomic features used in the study were extracted using MetaPhlAn. Although the tool is accurate with regard to the identified taxa, the relative abundance estimates did not account for unknown taxa and therefore might not be comparable across samples. This is especially concerning as infant nasal mWGS has not been previously studied. Can the authors assess the consistency of these feature vectors, to ones that took into account unidentified taxa (e.g. using the new MAG catalog PMID: 30661755 that some of the authors have participated in generating)?*

Many thanks for catching these aspects of our taxonomic profiling methods, which do vary from setting to setting. To ensure this did not affect our study’s results, we evaluated the robustness of the original findings to the influence of unknown taxa, especially given that this is the first use of shotgun metagenomic sequencing for the infant nasal microbiome. In brief, there was no substantial change in our significant findings when explicitly accounting for unidentified taxa.

In order to assess this, we rescaled the study’s taxonomic profiles produced by MetaPhlAn 2 by an estimate of the percentage of unknown sample mass. This percentage was calculated as the proportion of unmapped reads in HUMAnN 2 relative to the number of input reads (i.e. the KneadData 2 final read count). Next we used this proportion to directly rescale the taxonomic profiles; for example, if a sample was 50% species A and 50% species B via MetaPhlAn 2 and we calculated that 40% of reads were unmapped in HUMAnN 2, then our rescaled profile would be 30% species A, 30% species B, and 40% unknown.

We repeated the linear modeling using these rescaled profiles and the same methods as described originally in the manuscript, focusing on the new continuous variable “time point of first *S. aureus* acquisition” introduced above. In the results (**Fig. S7**), the main findings from the original study remained unchanged (i.e. associations with daycare, infant “ever” acquisition of *S. aureus,* and some associations with breastfeeding and infant *S. aureus* gain, and), with already-weak associations linked to breastfeeding and *S. aureus* gain dropping over the threshold of significance (q<0.25). Examination of the new results showed that these borderline associations consistently maintained the same direction of association and relative ranking among the results when sorted by q-value, but passed from slightly below to slightly above the critical value, as expected.

There was a high correlation (Spearman rho = 0.69, p<10^-15^ across all 208 filtered samples) between our measure of the proportion of human contamination (defined as one minus the proportion of reads after vs. before human depletion) and our measure of the proportion of unmapped sample mass. This suggests that this unknown, unmapped quantity was largely made up of cryptic human contamination. Among other covariates, our linear models were adjusted for the measure of the proportion of human contamination (defined as one minus the proportion of reads after vs. before human depletion). By rescaling the taxonomic profiles for this analysis, we effectively forced them to be influenced by the percentage of unmapped reads (a contamination signal that was not present in the taxonomic profiles originally due to the way they were normalized in MetaPhlAn 2). Since the linear models were already adjusted for human contamination, we greatly reduced model power by introducing this signal and then relatively crudely re-normalizing it out again via model adjustment. This was a useful sensitivity analysis to perform, and we’ve concluded that our original approach of using unadjusted MetaPhlAn 2 taxonomic profiles and adjusting linear models for the proportion of human contamination was a robust approach.

Based on these findings, we’ve updated the **Results** to explain the linear modeling results for *S. aureus* gain and included a new supplementary figure for the results of the analysis (**Fig. S7**, included below):

“In the taxonomic subset of these linear models, abundances of infant *Acinetobacter* species, *Streptococcus parasanguinis*, *Streptococcus salivarius*, and *Veillonella* species were weakly positively associated with *S. aureus* acquisition by the next month's sample (FDR q<0.25, **Fig. 3**). These trends tended to be driven by acquisitions occurring at later time points; presence of any of the four species was more frequently observed during acquisition events occuring after the first month (Fisher’s exact test, p=0.0805). The nasal microbiome may thus play a larger role in *S. aureus* carriage patterns as infants get older, which is concordant with a diminishing maternal and increasing environmental influence over time. However, we were limited in our cohort in the number of acquisition events occurring after the first month, causing the significance of these associations - except for *S. parasanguinis* - to be somewhat dependent on model specification (i.e. not always significant during robustness assessment, **Methods**, **Fig. S7**). Although this finding is exploratory in nature, we recommend that future studies account for early and late acquisitions events differently in order to ensure sufficient and balanced sample sizes.”

We’ve also updated the “Data processing and quality control” section of the **Methods** to describe this analysis:

“Due to the presence of quality-controlled DNA that didn’t map, we treated the unmapped sample mass like a microbial feature (i.e. rescaled the taxonomic profiles by the percent mapped reads and included a feature for the percent unmapped reads) and assessed the robustness of our results using these new profiles. The percent unmapped reads was strongly correlated with human contamination (Spearman rho = 0.69, p<10^-15^ across all 208 filtered samples) and didn’t change our main findings from linear models (**Fig. 3, Fig. S7**), other than reducing power (due to noise introduction and re-removal) and causing some weaker associations to drop out. We concluded that much of this unmapped sample mass represented mainly cryptic human contamination and that our initial approach of focusing purely on mapped reads and adjusting linear models for the proportion of human contamination was robust.”

##### **“Supplementary Figure 7: Significant associations between nasal microbiome taxonomic and subject phenotypes in a sensitivity analysis of the unmapped sample mass.** The unmapped sample mass was treated like a taxonomic feature (i.e. taxonomic profiles were rescaled by the percent mapped reads, and the percent unmapped reads was included as a feature), and linear models were rerun. Significant associations (q<0.25) between individual taxonomic features and phenotypic covariates using a MaAsLin multivariable linear model for the rescaled profiles are shown. An annotation of “+” indicates that the association was not seen in the original (**Fig. 3**) linear models, and an annotation of “-” indicates that the association was only seen in the original (**Fig. 3**) linear models. Overall, the percent unmapped sample mass was mostly representative of human contamination. As a result, model power was reduced by inducing a relationship between taxonomic composition and human contamination, and then also subsequently re-adjusting for human contamination as a linear model covariate (**Methods**), causing weaker associations to drop over the threshold of significance.”

*3. It is unclear to me how "strain" is defined in the study. It looks like a strain is defined by the significance of similarity (367-369, infants carry maternal strain because the gene content is significantly more similar than an unrelated mother), instead of the effect size of similarity (which is a more common definition). It is also questionable if similarity in gene content presence/absence (which is unlikely clock-like given the time scale) could infer transmission (365-366).*

“Strain” is notoriously difficult to define in microbes, particularly within communities, and in this manuscript we define it operationally to refer to microbial genome bins with the same genotype (i.e. genetically identical within the measurement limits of our assay). This definition is reflected in the paper by introducing the definition as “gene-content based strain profiling” and generally referring to it this way throughout (e.g. “gene-level strain profiles”). The exception is in the Discussion, where one paragraph discusses previous work using pulsed field gel electrophoresis- (PFGE) based *S. aureus* strain profiling.

Gene-content based or “phylogenomic” strain comparisons have been used for ~20 years now to identify either direct or recent transmission events (Eisen and Fraser 2003), particularly recently during the era of metagenomic assays(Human Microbiome Project Consortium 2012; Scholz *et al.* 2016; Yassour *et al.* 2018; Zeevi *et al.* 2019). No one gene difference need be clock-like in order to make this accurate. Instead, near-identical gene profiles are extremely unlikely to occur in random strains by chance, hence focusing on the similarity (not just significance) of gene content as a likely marker of direct or shared, indirect, recent transmission.

In particular, we calculated Jaccard distances (themselves a type of effect size) between *S. aureus* accessory gene profiles in related and unrelated mother-infant pairs (**Fig. 5D**, included below for convenience). Next, we used a one-sided Wilcoxon-Mann-Whitney on these two sets of genetic distances to determine whether distances were significantly lower than expected due to chance between infants and related mothers (where direct transmission could possibly occur) compared to infants and unrelated mothers (where direct transmission could not occur in this cohort).

The advantages of this approach for strain analysis included that we did not have to set a strict cut-off for determining if two variants were considered the same strain and our approach was robust to undersampling. Given the limited sequencing depth available after human nucleotide depletion in nasal samples, we expected undersampling (i.e. failing to identify a gene by sequencing that is truly present within a microbe in a sample) to increase the genetic distance between samples. However, we expected that this effect would occur equally for both related and unrelated mother-infant pairs; therefore, while the exact Jaccard distance for related mother-infant pairs is not easily interpretable due to undersampling, the difference between the related and unrelated groups is meaningful.

##### **“Figure 5: Gene-content based strain profiling for *S. aureus* and other common nasal species.** To perform gene-content based strain profiling for each species, pairwise Jaccard distances were calculated between samples on accessory gene presence/absence and averaged over same-subject pairs. Species/distance metric combinations with fewer than three subject pairs formed from six fully unique subjects are not displayed (see **Table S3** for sample sizes). Significance testing was performed with a one-sided Wilcoxon-Mann-Whitney test.”

*4. The authors suggested that infant strain gene contents are similar to their mothers', while 1) age-group adaptation could serve as a confounding factor, 2) infant microbiome is dynamic, 3) daycare has a strong influence on overall microbiome composition. In this case, does the gene content similarity between mother-infant pairs decrease overtime/correlate with daycare attendance?*

This is an interesting additional analysis suggestion, which was hinted at by some of the original tests, but which we have now carried out more thoroughly here. In brief, this didn’t produce any additional associations, but did re-confirm the joint relationship between time, daycare attendance (which tends to happen later during the first year), and microbiome development.

In more detail, our initial submission found that infants had gene content more similar to their own mothers compared to unrelated mothers for *S. aureus* and *C. acnes*, although the significance for *C. acnes* was mainly due to its very large sample size (it is abundant in many samples, and thus well-sequenced for genetic analysis)*.* Unfortunately, we don’t have comparable data to answer this question directly for *S. aureus*, since there were overall few related mother-infant pairs where *S. aureus* was both present and sufficiently abundant to be profilable with PanPhlAn. We had four related mother-infant pairs (8 total individuals) with *S. aureus* strain profiling data and one time point each per subject, for a total of 8 paired strain profiles.

However, we do not think these three factors are responsible for our findings, as they would all be expected to *reduce* the similarity between related mothers and infants. Additionally, mothers and infants likely have extended close contact with each other over the first year, so these external factors may not be as important for strain carriage (as opposed to abundance). Additionally, although our study was not designed to capture strain dynamics specifically, our findings are consistent with other studies on strains that are more targeted to be able to answer these questions (Dominguez-Bello *et al.* 2010; Nayfach *et al.* 2016; Ferretti *et al.* 2018; Yassour *et al.* 2018; Reiss-Mandel *et al.* 2019).

In order to explore this question to the extent possible with the available data, we have now performed an additional, similar analysis using more commonly detected species. In the manuscript, we identified gene family presence/absence in eight common nasal species using PanPhlAn, with the number of successfully profiled samples ranging from 20-98 per species. *C. acnes* and *D. pigrum* were both profilable in more than 25% of total samples (83 and 98 profiled samples, respectively) and thus were selected for this additional analysis. Furthermore, these species are biologically interesting because *C. acnes* is often dominant in the early nasal microbiome, while *D. pigrum* becomes a dominant microbiome member later in the first year after birth.

First, within species, we calculated the Jaccard distance between related mother-infant samples that were measured at the same time point and calculated the Spearman correlation between genetic distance and time point measured. Time measured and genetic distance were not strongly correlated for *C. acnes* (Spearman rho = 0.214, p=0.482 across 13 pairs), but were significantly inversely correlated for *D. pigrum* (Spearman rho = -0.753, p=0.031 across 8 pairs). For both species, just one pair had an infant who had attended daycare in the preceding month so we were not able to assess the effect of daycare. Next, within species, we calculated the Jaccard distance between all related mother-infant samples and calculated the Spearman correlation between genetic distance and the absolute value of the difference between infants’ and mothers’ time points. This did not produce any additional significant associations for *C. acnes* or *D. pigrum*.

As seen both here and in previous work (Sá-Leão *et al.* 2008; Teo *et al.* 2015; Ta *et al.* 2018), daycare plays a major role in shaping the early nasal microbiome. Therefore, further work exploring the effect of daycare not just on species acquisition, but strain replacement for existing species would be very interesting. Given our focus on *S. aureus*, we were not able to select samples for sequencing such that we could also assess strain replacement due to daycare attendance; however, we do not have reason to think that daycare attendance is confounding the results for *S. aureus* that we found in this study.

*5. (132-133) "..time points immediately preceding and following changes in S. aureus carriage status (were selected for sequencing)" does not seem to hold (for example, Row 4 in Fig. 1 - infant SA/early).*

We thank Reviewer #1 for pointing out this inconsistency, which arises due to our quality control procedures. In this example, the time points immediately preceding and following changes in *S. aureus* status were selected for sequencing for subject 556C (Row 4 in **Fig. 1**), but did not remain after host and read depth filtering. More generally, **Fig. 1** only shows sequencing as non-missing for samples that passed quality control. We have updated its caption and elsewhere in the manuscript (particularly **Methods**) to refer to the 208 samples used, instead of 284 samples originally selected for sequencing:

###### **Abstract**: “We recruited 36 mother-infant pairs and profiled a subset of monthly longitudinal nasal samples from the first year after birth (n=208 after QC) using shotgun metagenomic sequencing.”

###### **Results**: “This resulted in a total of 284 samples profiled metagenomically, with 208 samples passing sample quality control procedures (**Fig. 1**, **Methods**).”

###### **Results**: “Among retained samples, we sequenced 71, 43, and 30 samples from early, late, and never acquirer infants, respectively, as well as 13 samples from persistently colonized infants and 64 samples from mothers (**Fig. 1**).”

###### **Fig. 1** caption: “Culture testing for *S. aureus* was performed on all samples to assess carriage. Culture results, red for *S. aureus* positive and blue for *S. aureus* negative, are shown as the icon fill color. A subset of 208 samples were profiled with shotgun metagenomic sequencing and retained after quality control (**Methods**) for further analyses. Sequencing is shown as performed only for samples that passed QC procedures. Square icons indicate samples where sequencing was performed, and the outline color, red for *S. aureus* positive and blue for *S. aureus* negative, indicates whether *S. aureus* was identified by sequencing.”

*6. Panel labels (a/b/etc.) were not included for Fig. 3 and Fig. 4.*

Thank you, we have fixed this in both figures.

# Reviewer #2:

1. *The study has some minor shortcomings (see below), including the selection procedure of the samples ("time points immediately preceding and following changes in S. aureus carriage status as determined by culture test in infants") and the high drop-out rate of samples due to low quality sequencing results, resulting in some time points with only a few datasets available (e.g. for month 9, infant). This restriction impairs the overall consistency, but probably does not affect the results.*

We appreciate the reviewer’s time and input on the study, and their overall favorable comments; we’ve updated the manuscript to address the drawbacks called out here, with point-by-point responses below.

2. *As the authors state, this is one of the first reports to describe the development of the nasal microbiome of infants via metagenomics sequencing. Considering this, I would propose to include general information on the taxonomic and functional composition of the microbiome, and focus to Staphylococcus later in the manuscript.*

We agree with the reviewer about this point and thank them for their input. Since our study was designed around *S. aureus* (such as the selection of samples for sequencing), we were generally restricted to mainly focusing on these samples in which *S. aureus* was often dominant. Many additional samples were collected, though, that could be sequenced for a follow-up study on general microbiome development. However, throughout this study, we did aim to include many comparisons with previous 16S studies of the infant nasal microbiome and to investigate general developmental trends in the infants. This included exploring both alpha and beta taxonomic diversity in the infants over time (**Fig. 2**), as well as exploring the effects of daycare attendance and breastfeeding on the infant nasal microbiomes (**Fig. 3**) (antibiotic usage and delivery mode were recorded, but excluded from the analysis due to rarity, see below).

In comparison to previous 16S studies, which reported inconsistent alpha diversity trends in the nasal microbiomes of healthy infants, we found that alpha diversity increased over time. As expected, this parallels microbiome development across other body areas, particularly the gut (Koenig *et al.* 2011; Bäckhed *et al.* 2015). Our results also agreed with prior research that suggested that nasal microbiome development likely continues beyond one year - again similar to the rest of the body. We found that infants had taxonomic composition significantly more similar to their own mother than unrelated mothers at month one (and more generally at early time points, although this didn’t reach significance due to lower power). This is consistent with studies of the gut microbiome, but - as far as we’re aware - has not previously been reported for the nasal microbiome. Our findings for daycare were consistent with multiple previous 16S studies (Sá-Leão *et al.* 2008; Teo *et al.* 2015; Ta *et al.* 2018)and our findings for breastfeeding collaborated the findings of one prior 16S study (Biesbroek *et al.* 2014).

Generally, the taxonomic composition of our samples matched that seen in previous studies(Bomar *et al.* 2018; Kumpitsch *et al.* 2019). The most common genera in the infant nasal microbiome are reported to be *Moraxella*, *Streptococcus*, *Haemophilus*, *Staphylococcus*, and *Corynebacterium* with some studies also reporting high *Dolosigranulum* (Bomar *et al.* 2018; Kumpitsch *et al.* 2019). Combined, these genera (including *Dolosigranulum*) made up 71.78% of the total abundance of the infant samples. Additionally, they all had representatives among the top ten species (by mean abundance), which included: *Dolosigranulum pigrum* (30.60%), *Cutibacterium acnes* (18.44%, previously *Propionibacterium acnes*), *Moraxella catarrhalis* (6.06%), *Streptococcus mitis/oralis/pneumoniae* (6.03%), *Corynebacterium propinquum* (5.68%), *Staphylococcus aureus* (5.17%), *Staphylococcus epidermidis* (4.82%), *Corynebacterium accolens* (4.07%), *Propionibacteriaceae* unclassified (3.99%), and *Haemophilus influenzae* (3.86%). In contrast to some previous studies, we identified *Cutibacterium* (previously *Propionibacterium*) as a major genus dominating the infant nasal microbiome in the first year of life. This difference is likely due to previous studies often sequencing hypervariable region 4 of the 16S ribosomal RNA (rRNA) gene, which has been shown to severely underestimate *Propionibacterium* abundance (Meisel *et al.* 2016).

Strikingly, *Cutibacterium acnes* and Propionibacteriaceae unclassified (likely *C. acnes* that could not be identified due to low sequencing read counts) made up a combined 60.13% of the average relative abundance of infant samples at birth, while *Dolosigranulum pigrum* tended to dominate later (especially after daycare). In a linear model, birth time points were associated with significantly elevated *C. acnes* and Propionibacteriaceae unclassified (q<0.25), while later (non-birth) time points were associated with significant elevations of *D. pigrum, C. pseudodiphtheriticum*, *M. catarrhalis*, and *S. tigurinus* (q<0.25). Previous research has suggested that interaction with *C. acnes* can increase *S. aureus* virulence(Lo *et al.* 2011), which may help *S. aureus* to invade the human host (Lo *et al.* 2011). Similarly, the *C. acnes*-produced small molecule coproporphyrin III can promote *S. aureus* biofilm formation, which may improve *S. aureus* survival in hostile environments (Wollenberg *et al.* 2014). In our study, just over half of infants gained *S. aureus* at or before month one - it is possible that this low-diversity *C. acnes* dominated nasal microbiome at birth promotes or fails to prevent *S. aureus* acquisition, in contrast to more complex *D. pigrum*-dominated microbiome compositions seen later in the first year.

To better reflect this information in the manuscript, we’ve summarized it in a newly added **Discussion** paragraph:

###### Generally, the taxonomic composition of our samples matched that seen in previous studies (Bomar *et al.* 2018; Kumpitsch *et al.* 2019) with *Moraxella*, *Streptococcus*, *Haemophilus*, *Staphylococcus*, *Corynebacterium* and *Dolosigranulum* making up 71.78% of the total abundance of infant samples. In contrast to some previous studies, we identified *Cutibacterium* (previously *Propionibacterium*) as a major genus dominating the infant nasal microbiome in the first year of life. This difference is likely due to previous studies often sequencing hypervariable region 4 of the 16S ribosomal RNA (rRNA) gene, which has been shown to severely underestimate *Propionibacterium* abundance (Meisel *et al.* 2016). After *Dolosigranulum pigrum* (30.60%), *Cutibacterium acnes* had the second highest mean abundance among infants (18.44%). In a linear model, birth time points were significantly (q<0.25) associated with *C. acnes* and *Propionibacteriaceae* unclassified (possibly *C. acnes* that could not be identified due to low sequencing read counts), which together made up 60.13% of the relative abundance of samples at birth. *D. pigrum* tended to dominate subsequently, and later (non-birth) time points were associated with significant elevations of *D. pigrum, C. pseudodiphtheriticum*, *M. catarrhalis*, and *S. tigurinus* (q<0.25). Previous research has suggested that interaction with *C. acnes* can increase *S. aureus* virulence (Lo *et al.* 2011), helping *S. aureus* to invade the human host (Lo *et al.* 2011). Similarly, the *C. acnes*-produced small molecule coproporphyrin III can promote *S. aureus* biofilm formation, potentially improving its survival in challenging environments (Wollenberg *et al.* 2014). In our study, just over half of infants gained *S. aureus* at or before month one; in contrast to more complex *D. pigrum*-dominated microbiome compositions seen later in the first year, this low-diversity *C. acnes* dominated nasal microbiome at birth may fail to prevent or even promote *S. aureus* acquisition.

3. *Also with respect to the viral/phage load, the sample information could be exploited more. As Staphylococcus phages are largely abundant in nasal samples, I consider they might also influence the nasal microbial community substantially. Viral/phage information could be obtained from the metagenomics dataset without large effort, and the derived results could be included in the manuscript.*

Reviewer #2 raises a thoughtful point regarding the role of viral/phage load on *S. aureus* carriage. In general, phage and eukaryotic viruses are only variably detectable in typical (non-enriched) metagenomic profiles, due to their unique extraction and isolation characteristics. Here, 28 viral features were identified in at least one sample by MetaPhlAn, including six *Staphylococcus* phages (*Staphylococcus phage 3A*, *Staphylococcus phage 80alpha*, *Staphylococcus phage PVL*, *Staphylococcus phage phiETA*, *Staphylococcus phage phiETA2*, *Staphylococcus phage tp310-2*). All detected viruses were included in our previous analysis, but did not result in any significant associations, and are thus not emphasized in the text.

To investigate them in more detail, the filtered taxonomic profiles with viruses included:

First, we calculated simple, direct Spearman correlations between *S. aureus* abundance and the abundance of each viral feature in mothers and infants (separately). No viral features were significantly correlated with *S. aureus* in mothers or infants (all q>0.25).

Next, we reran our main analyses including viral features. We followed the same feature filtering procedure as described in the methods: all filtering was applied separately to infant and mother samples due to the differences in microbiome composition, and all feature abundances below 0.1% (taxonomy) in more than 95% of samples were aggregated to a single “other” category. Of 28 total viral features, three remained after filtering (*Propionibacterium phage P101A*, *Propionibacterium phage P14-4*, *Dasheen mosaic virus*) in the infants, and five in mothers (*Propionibacterium phage P100D*, *Propionibacterium phage P101A*, *Staphylococcus phage 80alpha*, *Staphylococcus phage PVL*, *Dasheen mosaic virus*). In linear modeling, none of the viral features were significantly (q<0.25) associated with *S. aureus* phenotype variables or other covariates.

Next, we first grouped common phage (*Propionibacterium* and *Staphylococcus* phage) abundance into one “taxon” each and repeated the above analyses. Both combined features survived feature filtering. In linear models, neither of the combined viral features was significantly (q<0.25) associated with *S. aureus* phenotype variables or other covariates. Due to the lack of significant results, we did not repeat the random forest modeling with these features. We’ve updated the “Data processing and quality control” section of the **Methods** to reference this analysis:

###### “Phage abundance was originally included in the taxonomic profiles, but due to the lack of any significant associations (q<0.25) in linear models, phage abundance was normalized out and subsequent analyses focused purely on non-viral taxa.”

*Line 43 and elsewhere: it is true, 284 samples were sequenced, but only 208 samples were retained for further analyses. Please correct throughout, as mentioning the higher number is somewhat misleading.*

We thank Reviewer #2 for pointing out this inconsistency, which Reviewer #1 also caught. We introduced our fixes for it above, which throughout the manuscript consist of:

###### **Abstract**: “We recruited 36 mother-infant pairs and profiled a subset of monthly longitudinal nasal samples from the first year after birth (n=208 after QC) using shotgun metagenomic sequencing.”

###### **Results**: “This resulted in a total of 284 samples profiled metagenomically, with 208 samples passing sample quality control procedures (**Fig. 1**, **Methods**).”

###### **Results**: “Among retained samples, we sequenced 71, 43, and 30 samples from early, late, and never acquirer infants, respectively, as well as 13 samples from persistently colonized infants and 64 samples from mothers (**Fig. 1**).”

###### **Fig. 1** caption: “Culture testing for *S. aureus* was performed on all samples to assess carriage. Culture results, red for *S. aureus* positive and blue for *S. aureus* negative, are shown as the icon fill color. A subset of 208 samples were profiled with shotgun metagenomic sequencing and retained after quality control (**Methods**) for further analyses. Sequencing is shown as performed only for samples that passed QC procedures. Square icons indicate samples where sequencing was performed, and the outline color, red for *S. aureus* positive and blue for *S. aureus* negative, indicates whether *S. aureus* was identified by sequencing.”

*Page numbers are missing in pages before line 166.*

*Spaces are missing before the brackets for the references throughout the manuscript.*

*Line 86: correct: "Esp that that inhibits"*

Thank you, we have fixed these errors.

*Line 94 to 108: This chapter could be condensed, as some of the information is partially redundant.*

We agree, and we have shortened this paragraph to read:

###### “Understanding the relationship between the nasal microbiome and *S. aureus* carriage in early life is especially important because the infant microbiome across the body undergoes dramatic shifts in the first year (Koenig *et al.* 2011; Maynard *et al.* 2012), and infants have also been shown to acquire and lose *S. aureus* in the nares during this time (Lebon *et al.* 2008; Leshem *et al.* 2012). Prior studies of *S. aureus* carriage have mainly been performed in adults and are not necessarily generalizable to infants, as the infant microbiome differs greatly from that of adults during this early period of rapid development (Capone *et al.* 2011; Bomar *et al.* 2018). Infant nasal microbiome colonization may be influenced by many of the same environmental factors as the gut microbiome (e.g. delivery mode, feeding method, antibiotic usage, and siblings (Bomar *et al.* 2018; Ta *et al.* 2018; Rawls and Ellis 2019)), but it has been substantially less studied than has the gut (Peterson *et al.* 2016; Brugger *et al.* 2016; Rawls and Ellis 2019). Particularly with the goal of mitigating later *S. aureus* infection risk, additional longitudinal studies in this area can determine whether microbiome changes causally precede *S. aureus* acquisition or loss and shed light on the development of the infant nasal microbiome, which is also broadly involved in later asthma, allergies, and upper respiratory tract infections (Mika *et al.* 2015; Shilts *et al.* 2016; Proctor and Relman 2017; Ta *et al.* 2018).”

*Line 147: can you explain the large difference between mean and median?*

This large difference between the mean and median occurs because there are a number of culture-positive samples where *S. aureus* dominates the microbiome. In **Fig. S1A** (quoted below for convenience), among the samples with a positive *S. aureus* culture test, the median is very low (almost zero), but there are outliers with very high *S. aureus* abundance relative abundance, which increase the mean. The difference between the mean and median is not inconsistent, and that is why we chose to report both values.

##### **“Supplementary Figure 1: *S. aureus* test concordance. (a)** Detection of *S. aureus* by culture and sequencing showed strong, but not complete, concordance, due to high rates of false negatives. **(b)** Sequencing false negatives (i.e. samples positive for *S. aureus* by culture, but with zero *S. aureus* relative abundance by sequencing) tended to have overall lower sample read depth compared to sequencing true negatives.”

*Line 150-151: this explanation is very vague; you would expect the sequencing approach to be more sensitive and to also detect non-cultivable ones; maybe extend the explanations a bit.*

We agree that the nuance is a bit difficult to convey here, since sequencing is often considered to be more sensitive than culture, but in many cases the exact opposite is true. For *S. aureus*, for example, since its culture conditions have been so well-studied, extremely low-abundance colonizations can be recovered by culture (e.g. one cell in a million) that are not detectable by typical metagenomics. Conversely, for other organisms that are less readily culturable, sequencing can detect them when sufficiently abundant, even if they are difficult to grow in culture.

In this case, we are comparing the performance of sequencing and culture for the identification just of one specific microbe, *S. aureus*, instead of nasal microbiome composition more generally. Adding complexity, *S. aureus* has been shown to enter a viable but non-culturable (VBNC) state in biofilms and with antibiotic exposure (Li *et al.* 2014). In this state, it is not culturable on routine agar, but has undamaged genetic information that could be sequenced. Additionally, previous research found that the sensitivity of culture for the detection of *S. aureus* is highly positively correlated with *S. aureus* absolute abundance (Liu *et al.* 2015). This suggests that culture-based methods may sometimes - but, unlike sequencing, not always - fail to identify *S. aureus* when it is present only at a low quantity.

Conversely, other microbes identified by culture can also fail to show up in sequencing if the microbe is insufficiently resolved (i.e. differentiated from other similar species). For example, *S. aureus* and *S. epidermidis* have closely related genomes that can be difficult to tell apart, especially by amplicon sequencing and sometimes even metagenomically. In **Fig. 3**, the abundance of *S. epidermidis* in infants is weakly positively associated with the identification of *S. aureus* by sequencing in infants, likely due to occasional misclassification of reads between the two species. More simply, when *S. aureus* is present in a sample at low abundance, it may be missed by sequencing (like any other microbe) if the sample has a low read count. In **Fig. S1** (included below for convenience), samples where *S. aureus* was identified in culture, but not sequencing tended to have a lower read depth, typically due to the high amount of human DNA carried in nasal swab samples.

In order to briefly summarize this background information in the manuscript, we’ve added **Results** text reading:

###### “...may miss a significant fraction of *S. aureus* carriage (Liu *et al.* 2015), in part due to idiosyncratic mechanisms such as viable but non-culturable (VBNC) biofilm formation (Li *et al.* 2014). Conversely, any sufficiently rare microbe - including *S. aureus* *-* can be missed by sequencing of insufficient depth (which in sites such as the nares includes human nucleotides; **Fig. S1**). Due to these likely false negatives from both assays, our subsequent...”

##### **“Supplementary Figure 1: *S. aureus* test concordance.** (a) Detection of *S. aureus* by culture and sequencing showed strong, but not complete, concordance, due to high rates of false negatives. (b) Sequencing false negatives (i.e. samples positive for *S. aureus* by culture, but with zero *S. aureus* relative abundance by sequencing) tended to have overall lower sample read depth compared to sequencing true negatives.”

*Fig. 1: I propose to split the figure into two panels, one for the culture results, one (next to it) for the sequencing result; the grey rims of the boxes are very difficult to identify. Extend the Figure legend and mention explicitly that the carriage (title of the legend) was analyzed via cultivation. Explain the colors also in the legend. Number the infant-mother pairs (each row).*

We are grateful to Reviewer #2 for this helpful feedback on making **Fig. 1** clearer, which also relates to the number of samples retained after quality control. We have updated the figure to use a circle with no border for non-sequenced samples to avoid the use of the grey rims entirely, and we added numbers for each row/mother-infant pair. Additionally, we’ve updated the caption to mention that carriage was assessed via cultivation and to explain the colors:

“**Figure 1: Longitudinal shotgun metagenomics of the nasal microbiome and *Staphylococcus aureus* carriage in 36 mother-infant pairs.** Nasal swabs were taken from 36 mother-infant pairs at birth and monthly for the first year after birth. Culture testing for *S. aureus* was performed on all samples to assess carriage. Culture results, red for S. aureus positive and blue for *S. aureus* negative, are shown as the icon fill color. A subset of 208 samples were profiled with shotgun metagenomic sequencing and retained after quality control (**Methods**) for further analyses. Sequencing is shown as performed only for samples that passed QC procedures. Square icons indicate samples where sequencing was performed, and the outline color, red for *S. aureus* positive and blue for *S. aureus* negative, indicates whether *S. aureus* was identified by sequencing. Infants displayed three unique patterns of *S. aureus* carriage highlighted here and used for sequenced sample prioritization: "Early" (acquisition by month one), "Late" (acquisition after month one, also typically transient), and "Never" (no acquisition during the study period).”

*Fig. 2: I propose to show the same resolution of data also for mothers (for those time points that are possible) in Fig. 2A, in order to visualize the variability also in this dataset. In Fig. 2B1: please explain the line at timepoint 0. In Fig. 2B3: highlighting of the significance now refers also to Fig. 1b1, so maybe indicate not associated to the sampling months but differently.*

We thank Reviewer #2 for these thoughtful ideas regarding **Fig. 2**. All mothers had sequencing performed at birth, month one, and then one later time point (matched to a later time point for their infant, making this not the same time point for all mothers). We have updated **Fig. 2A** to show more detail for the mothers; specifically, we show alpha diversity at birth, month one, and a combined “other/later time point”. There were 19 data points for mothers at birth, 24 at month one, and 21 at later time points. We chose to display the data this way because among later time points, only month 7 had five or more data points.

When the data was split up this way, the later time point had significantly lower alpha diversity compared to month one (p = 0.0136, one-sided Wilcoxon rank sum test), but was not significantly different from the birth time point, nor were birth and month one time points significantly different from each other. For **Fig. 2B3**, we thus continue to use the comparison of infants against the average of all time points for their mother.

For **Fig. 2B1**, we calculated the Bray-Curtis distance between each infant’s taxonomic composition at the current time point (shown on the x-axis) and their composition at birth. At month 0 (birth), these compositions are identical and thus result in a Bray-Curtis distance of 0 for all infants, creating the appearance of a line on the plot. In response to this feedback, we have removed the line from the plot, replaced it with the label “NA” and added text to the caption to explain that by definition this quantity is not meaningful.

Finally, to correct the significance annotations in **Fig. 2B3** and make the plot more readable, we have given **Fig. 2B1-2** a separate x-axis. Furthermore, we have updated the **Results** to note that the trend of infants being more similar to their related mother than to unrelated mothers was significant at month one (where we had more samples and were more well-powered to detect it), but was also a more general trend early in time, although we were not as well powered to detect it at other months:

###### “Infants were significantly more similar to their own mother than to unrelated mothers at month one, but not at other time points (Wilcoxon-Mann-Whitney test, p=0.0026); this trend was generally true of early months, although it did not otherwise reach significance (**Fig. 2**). This supports the hypothesis that maternal influence on nasal microbiome composition mainly occurs at the earliest time points after birth. It is also consistent with studies on the infant gut microbiome, which find greater similarity between vaginally-delivered infants and their mothers shortly after birth, but that this similarity diminishes afterwards, often quickly, based on environmental exposures (Dominguez-Bello *et al.* 2010; Ferretti *et al.* 2018; Vatanen *et al.* 2018, 2019).”

##### “**Figure 2: Development of infant nasal microbiome composition over the first year of life. (a)** Infant nasal microbiome development showed general ecological patterns similar to those of other body sites. Alpha diversity increased over time in infants, although confidence was lower at later time points due to fewer samples; it decreased slightly at later time points (L) in mothers. Mothers were sequenced at birth, month one, and one later time point (matched to a later time point for their infant, making this not the same time point for all mothers), which is labeled as “L”. **(b)** Mean Bray-Curtis dissimilarity per time point comparing (1) infants to themselves at birth, (2) infants to themselves at their most recent preceding time point, (3) infants to related and unrelated mothers, (4) infants to other infants with the same or different *S. aureus* status at the same time point. In (1) the Bray-Curtis dissimilarity at birth is equal to 0 since we are comparing identical compositions and is labeled as “NA”. The use of the most recently recorded preceding time point for (2) is conservative because we have less frequent sampling at later time points, but still find that infants are more self-conserved at these times. *S. aureus* status as positivity by either sequencing or culture is shown here, but there were no group differences if *S. aureus* was defined using other phenotypes. In both graphs, time points with fewer than 5 data points are not displayed (see **Table S3** for sample sizes), and diversity measures are calculated on average subject taxonomic composition for the mothers (due to the limited sample numbers).”

*Line 205: Check sentence ("if"missing?)*

Thank you for this recommendation. We have rephrased this sentence to:

###### “To identify if individual microbial community features correlated with *S. aureus* status, we fit linear mixed models for the association between *S. aureus* phenotype and microbial features (taxonomic and functional) for infants and mothers separately, as well as for the association between infant phenotypes and the related mother’s microbial features (**Table S4**).”

*Line 228: "This is interesting" - please rephrase*

We have changed this to:

###### “However, the maternal abundance of the commensal species *Dolosigranulum pigrum* was inversely associated with infant “ever” acquisition of *S. aureus*. A number of epidemiological studies have reported an inverse association between *D. pigrum* and *S. aureus* in adults (Yan *et al.* 2013; Liu *et al.* 2015; Escapa *et al.* 2018; Brugger *et al.* 2019), as well as infants (Khamash *et al.* 2019), although the potential mechanisms or causality of the relationship is unknown”.

*Line 230 ff: This part is largely discussion and could be moved to the discussion section.*

We agree, and we’ve shortened the **Results** text to read simply:

###### “However, the maternal abundance of the commensal species *Dolosigranulum pigrum* was inversely associated with infant “ever” acquisition of *S. aureus*. A number of epidemiological studies have reported an inverse association between *D. pigrum* and *S. aureus* in adults (Yan *et al.* 2013; Liu *et al.* 2015; Escapa *et al.* 2018; Brugger *et al.* 2019), as well as infants (Khamash *et al.* 2019), although the potential mechanisms or causality of the relationship is unknown.”

We moved the more detailed information to the corresponding section of the **Discussion**:

###### “One of the other most striking observations in our data was the presence of a gene family, correlated with *D. pigrum* genomic content, that was strongly co-exclusionary with *S. aureus* carriage. Due to its similarity with 16S rRNA gene sequences, this gene family likely represents a species not closely homologous to current reference isolates that competes with *S. aureus*. Its correlation with *D. pigrum* in our dataset suggests that it may either occupy a related phylogenetic space, or that these two species may act as co-colonizers. Furthermore, previous epidemiological studies have reported inverse associations between *D. pigrum* and *S. aureus* in adults (Yan *et al.* 2013; Liu *et al.* 2015; Escapa *et al.* 2018; Brugger *et al.* 2019) and infants (Khamash *et al.* 2019), and identified diverse biosynthetic gene clusters in *D. pigrum* believed to be involved in its in vitro inhibition of *S. aureus* (Brugger *et al.* 2019). In one study of hospitalized neonates, cases (those acquiring *S. aureus*) had lower *D. pigrum* relative abundance compared to matched controls seven days prior to *S. aureus* acquisition (Khamash *et al.* 2019), and a recent study found that 10 of 10 diverse *D. pigrum* isolates inhibited *S. aureus* growth in vitro (Brugger *et al.* 2019). The mechanisms through which *D. pigrum* inhibited *S. aureus* in this experiment were not fully clear, but one hypothesis explored by the authors was an enrichment of biosynthetic gene clusters (BGCs) in *D. pigrum*, with strains containing both a lanthipeptide BGC and a bacteriocin inducing the greatest inhibition (Brugger *et al.* 2019). Our finding of the inverse association between maternal *D. pigrum* and infant “ever” acquisition of *S. aureus*, however, is the first to suggest that *D. pigrum* is one pathway through which the maternal microbiome influences infant *S. aureus* carriage. From our results alone, it is not clear whether *D. pigrum* prevents the acquisition of maternal *S. aureus* that could be passed to the infant, or whether maternal *D. pigrum* is passed to the infant where it is protective against *S. aureus* acquisition, and in either case whether *D. pigrum* itself or an uncharacterized relative are responsible for the additional sequences co-exclusionary with *S. aureus*.”

*Fig. 3: a) and b) should be included in the figure.*

Thank you, we have fixed this in **Fig. 3**.

*Line 292: please specify: not assigned to any level of taxonomy?*

HUMAnN 2, which was used for this analysis, only assigns genes (and thus pathways and other functional features) to taxonomy if they can be matched as nucleotides to a specific species pan-genome. If matches occur based on e.g. amino acid homology instead, a gene is assigned to “unclassified”. To clarify this, we have changed this to:

###### “The functional feature most responsible for this improvement was gene family UniRef90 X5NU12, a small 42-amino acid sequence to which taxonomy was not assigned by our analysis.”

*Line 319/20: significantly should only be used in case of a statistically significant finding; so maybe rephrase*

We agree, and in this case we mean that the AUC of the random forest model had a 95% bootstrap confidence interval with a lower bound above 0.5. We’ve clarified this point in the manuscript by updating the sentence to:

###### “In total, 11 out of 13 of these models had AUCs that were significantly above 0.5 when using species as predictors (**Fig. 4)**, and 9 out of 13 models had AUCs significantly above 0.5 when using functions (**Fig. 4**, bootstrap 95% CI > 0.5).”

*Fig. 4: a) and b) should be included in the figure.*

Thank you, we have fixed this in **Fig. 4**.

*Line 431: "per se" should be in italics*

We have fixed this.

*Line 443: similarity with 16S rRNA sequences*

We have updated this to: “Due to its similarity with 16S rRNA gene sequences”. We also updated the following **Results** sentence to:

###### “A BLAST (66) search of reads mapping to this gene family revealed many significant alignments with 16S rRNA gene sequences nominally assigned to species as diverse as *Streptococcus*, *Staphylococcus*, *Lactobacillus, Cutibacterium,* and *Bifidobacterium*.”

*Line 444: completely uncharacterized? Not even on domain level?*

Our apologies for the miswording, which was intended to have the same meaning as above: our particular analysis workflow was unable to assign confident taxonomy to the sequence, and it was not sufficiently homologous to any available references to do so manually. To clarify this, instead of referring to a “taxonomically uncharacterized species”, we have this to read:

###### “Due to its similarity with 16S rRNA gene sequences, this gene family likely represents a species not closely homologous to current reference isolates that competes with *S. aureus.*”

*Line 470: this is a very important point: only mothers were included in the study, but also the other parent and siblings could contribute substantially; also skin contact during breast-feeding… consider to extend this part*

This is an excellent point; our **Results** directly include an analysis of breastfeeding already, and we have updated the additional **Discussion** text to read:

###### “Lastly, *S. aureus* carriage is a complex phenomenon, with ecological and microbiological variation undoubtedly contributed by unmeasured factors (e.g. immune development, contact with other caregivers and relatives, diet, etc.), and therefore microbiome contributions need to be large to be detectable. Particularly during highly dynamic infant colonization, a wide range of environmental influences (in addition to direct maternal effects) are likely to influence microbiome composition. These effects are further confounded since the mother’s nasal (and other) microbiome composition may in turn be influenced by some of these same factors due to shared genetics, cohabitation, lifestyle factors, and/or transmission of strains with other children, all of which have been demonstrated for the gut microbiome (Yatsunenko *et al.* 2012; Song *et al.* 2013; Goodrich *et al.* 2014; Bäckhed *et al.* 2015; Browne *et al.* 2017; Ferretti *et al.* 2018; Yassour *et al.* 2018; Brito *et al.* 2019). Through an integrative...”

*Line 499 ff: please identify inclusion and exclusion criteria for the cohort*

There were few inclusion and exclusion criteria for this study. The inclusion criteria were being a pregnant woman, attending the Sheba Medical Center obstetrics monitoring unit between 2013 to 2016, being at least 34 weeks pregnant, visiting the monitoring unit during screening hours (held for three hours per week), and providing consent to participate. Exclusion criteria included delivery prior to 34 weeks of pregnancy and failure to provide consent to participate. We have updated the “Cohort and experimental design” section of the **Methods** to state these more explicitly:

###### “More specifically, subjects were recruited from among pregnant women who attended the Sheba Medical Center obstetrics monitoring unit between 2013 to 2016, were at least 34 weeks pregnant, visited the monitoring unit during screening hours (held for three hours per week), and provided consent to participate. Exclusion criteria included delivery prior to 34 weeks of pregnancy and failure to provide consent to participate.”

*Line 520: please explain how the DNA was extracted; were controls included?*

We thank Reviewer #2 for pointing out this oversight. We have updated the “DNA extraction and sequencing” section of the **Methods** to include this information:

###### “DNA extraction was performed with the QIAGEN DNeasy PowerLyzer PowerSoil Kit with a bead beating protocol. After extraction, DNA concentration was measured by NanoDrop and frozen at -20°C until being shipped to the United States for sequencing. Prior to starting the project, the DNA extraction method was validated by performing extractions on double-distilled water (DDW) samples and confirming that no DNA concentration was measured by NanoDrop and that the displayed band was a true band and not due to contamination. Negative controls were not additionally sequenced since specimen DNA concentrations were measured after extraction to be reliably high and differentiated from potential background contaminants (due to the otherwise substantial added cost).”

*Line 572: title should be changed, as it should refer to a methodological approach*

Thank you for pointing this out. We’ve updated the title to: “Statistical methods for assessing infant developmental trends”.

*Line 589 and elsewhere: the dataset was frequently filtered using certain cut-offs. I would be interested to know whether other cut-offs have been tested, or this was based on previous experience/ reference information? Please clarify.*

We thank Reviewer #2 for pointing this out. These QC parameters do generally reflect our and similar studies’ best practices, the main rationale being to avoid testing features or covariates that are too uncommon to possibly detect an effect. That is, if a taxon is only nonzero in one or two samples, there’s no point in trying to correlate it with a phenotype. For example, microbiome features were filtered such that features with abundance below 0.1% (taxonomy) or 0.05% (function) in more than 95% of samples were aggregated to a single “other” category, and linear model covariates were only included in the model if the covariate was present in at least 10% of samples. This is not to say that these features or covariates are not inherently interesting, but they were not able to be tested in our dataset because they were too rare to be individually significant. We have updated the **Methods** text to better reflect this rationale for filtering:

###### “Prior to the creation of linear and random forest models, we filtered taxonomic and functional features to retain analyzable subsets of these features passing quality control. All filtering was applied separately to infant and mother samples due to the differences in microbiome composition, and all cut-points for filtering were selected *a priori* based on best practices from similar studies. First, all feature abundances below 0.1% (taxonomy) or 0.05% (function) abundance in more than 95% of samples were aggregated to a single “other” category to avoid testing features that were too uncommon to possibly detect an effect.”

###### “For mothers and infants separately, we included covariates in the model when (1) the covariate was significantly (q<0.25) associated with at least one species in a univariate prescreen, and (2) the covariate was at present in at least 10% of samples (including those that were missing covariate data). Furthermore, models for maternal microbiome features were not adjusted for variables considered primarily related to infants, such as breastfeeding and daycare. These criteria were decided upon *a priori*. The rationale for (2) was that these covariates were not able to be tested in our dataset because they were too rare to be individually significant.”

Analogously, several other QC filters were used for other analyses where there was not enough data to get a reliable answer. For example, this included only running random forest models if there were at least 20 samples in each class (sufficient for meaningful cross-validation). Additionally, the **Fig. 5** strain analysis comparisons were only displayed if they had data from at least three unique pairs consisting of six unique subjects (to avoid bias that could occur if many pairs were created with the same subject and this subject happened to have unusual data). **Supplementary Table 3** “Sample sizes for boxplots” gives the number of data points per comparison, and we’ve added **Supplementary Table 8**, which provides the number of data points per class for the random forest models. Overall, this filtering only served to exclude possibly unreliable results (but not to include any additional results), and the results that would drop out with different filtering levels can be observed in the supplementary tables. We have updated correspondingly updated the text to read:

###### “Random forest models were only run if they had at least 20 samples in each class (i.e. a sufficient size for meaningful cross-validation).”

Finally, many of these cut-offs were selected *a priori* based on previous papers or lab experience, biological rationale, recommended defaults of the programs we used, and common statistical practices. For example, during quality control, samples were retained in the analysis if they contained more than 5 x 10^4^ sequenced read pairs (mean 9.47 x 10^5^ read pairs per sample). This cut-point was based on approximately 1x coverage of an *E. coli* equivalent genome size, meaning that a microbe with a roughly *E. coli* sized genome would be expected to have, on average, barely complete coverage of its genome at our depth. We have updated the text to reflect this:

###### “Samples were retained in the analysis if they contained more than 5 x 10^4^ sequenced read pairs (mean 9.47 x 10^5^ read pairs per sample) and were not taxonomically 100% unclassified (detailed below). This cut-point was selected *a priori* based on approximately 1x coverage of an *E. coli* equivalent genome size.”

Similarly, the definition of *S. aureus* persistent carriage was defined *a priori* to match definitions used in the parent cohort study (Reiss-Mandel *et al.* 2019), but we also included a continuous version of this variable called “percent positive time points”. In response to this feedback, we have also created a continuous version of *S. aureus* early acquisition, as this was another *S. aureus* phenotype variable that relied on a cut-point. We created a subject-fixed *S. aureus* phenotype calculated using the *S. aureus* culture data that was defined as the first time point for which an infant tested positive (or NA for those subjects who never tested positive). We found that this variable was not associated with any taxonomic or functional features in the infants, which is very similar to the results for the binary *S. aureus* early acquisition variable. Due to the lack of significant results, this variable was not included in the random forest modeling.

We have updated **Supplementary Table 2**, **4**, and **5** to include information on this variable and the modeling results for this variable. Additionally, we updated the “*S. aureus* phenotype definitions” section of **Methods** as follows to include a description of the variable:

###### “The *S. aureus* phenotype of a sample was defined using multiple measures to capture different dimensions of *S. aureus* carriage. Subject-fixed *S. aureus* phenotypes were defined strictly by the *S. aureus* culture data, and included *S. aureus* early acquisition, late acquisition, “ever” acquisition, persistent carriage, the percent of positive time points, and the time point of first *S. aureus* acquisition...For infants, the time point of first *S. aureus* acquisition was defined as the first time point for which the subject tested positive for *S. aureus* or NA for subjects who never tested positive.”

Overall, we have updated the text of the manuscript to more fully explain our rationale for filtering, and to provide the reader with additional information to assess the full effects of quality control on our results.

**References**

Bäckhed F., J. Roswall, Y. Peng, Q. Feng, H. Jia, *et al.*, 2015 Dynamics and Stabilization of the Human Gut Microbiome during the First Year of Life. Cell Host Microbe 17: 852.

Biesbroek G., A. A. T. M. Bosch, X. Wang, B. J. F. Keijser, R. H. Veenhoven, *et al.*, 2014 The impact of breastfeeding on nasopharyngeal microbial communities in infants. Am. J. Respir. Crit. Care Med. 190: 298–308.

Bomar L., S. D. Brugger, and K. P. Lemon, 2018 Bacterial microbiota of the nasal passages across the span of human life. Curr. Opin. Microbiol. 41: 8–14.

Brito I. L., T. Gurry, S. Zhao, K. Huang, S. K. Young, *et al.*, 2019 Transmission of human-associated microbiota along family and social networks. Nat Microbiol 4: 964–971.

Browne H. P., B. A. Neville, S. C. Forster, and T. D. Lawley, 2017 Transmission of the gut microbiota: spreading of health. Nat. Rev. Microbiol. 15: 531–543.

Brugger S. D., L. Bomar, and K. P. Lemon, 2016 Commensal-Pathogen Interactions along the Human Nasal Passages. PLoS Pathog. 12: e1005633.

Brugger S. D., S. M. Eslami, M. M. Pettigrew, I. F. Escapa, M. M. Henke, *et al.*, 2019 Dolosigranulum pigrum cooperation and competition in human nasal microbiota. bioRxiv 678698.

Capone K. A., S. E. Dowd, G. N. Stamatas, and J. Nikolovski, 2011 Diversity of the human skin microbiome early in life. J. Invest. Dermatol. 131: 2026–2032.

Dominguez-Bello M. G., E. K. Costello, M. Contreras, M. Magris, G. Hidalgo, *et al.*, 2010 Delivery mode shapes the acquisition and structure of the initial microbiota across multiple body habitats in newborns. Proc. Natl. Acad. Sci. U. S. A. 107: 11971–11975.

Eisen J. A., and C. M. Fraser, 2003 Phylogenomics: intersection of evolution and genomics. Science 300: 1706–1707.

Escapa I. F., T. Chen, Y. Huang, P. Gajare, F. E. Dewhirst, *et al.*, 2018 New Insights into Human Nostril Microbiome from the Expanded Human Oral Microbiome Database (eHOMD): a Resource for the Microbiome of the Human Aerodigestive Tract. mSystems 3. https://doi.org/10.1128/mSystems.00187-18

Ferretti P., E. Pasolli, A. Tett, F. Asnicar, V. Gorfer, *et al.*, 2018 Mother-to-Infant Microbial Transmission from Different Body Sites Shapes the Developing Infant Gut Microbiome. Cell Host Microbe 24: 133–145.e5.

Goodrich J. K., J. L. Waters, A. C. Poole, J. L. Sutter, O. Koren, *et al.*, 2014 Human genetics shape the gut microbiome. Cell 159: 789–799.

Human Microbiome Project Consortium, 2012 Structure, function and diversity of the healthy human microbiome. Nature 486: 207–214.

Khamash D. F., E. F. Mongodin, J. R. White, A. Voskertchian, L. Hittle, *et al.*, 2019 The Association between the Developing Nasal Microbiota of Hospitalized Neonates and Staphylococcus aureus Colonization. Open Forum Infect Dis. https://doi.org/10.1093/ofid/ofz062

Koenig J. E., A. Spor, N. Scalfone, A. D. Fricker, J. Stombaugh, *et al.*, 2011 Succession of microbial consortia in the developing infant gut microbiome. Proc. Natl. Acad. Sci. U. S. A. 108 Suppl 1: 4578–4585.

Kumpitsch C., K. Koskinen, V. Schöpf, and C. Moissl-Eichinger, 2019 The microbiome of the upper respiratory tract in health and disease. BMC Biol. 17: 87.

Lebon A., J. A. M. Labout, H. A. Verbrugh, V. W. V. Jaddoe, A. Hofman, *et al.*, 2008 Dynamics and determinants of Staphylococcus aureus carriage in infancy: the Generation R Study. J. Clin. Microbiol. 46: 3517–3521.

Leshem E., A. Maayan-Metzger, G. Rahav, M. Dolitzki, J. Kuint, *et al.*, 2012 Transmission of Staphylococcus aureus from mothers to newborns. Pediatr. Infect. Dis. J. 31: 360–363.

Li L., N. Mendis, H. Trigui, J. D. Oliver, and S. P. Faucher, 2014 The importance of the viable but non-culturable state in human bacterial pathogens. Front. Microbiol. 5: 258.

Liu C. M., L. B. Price, B. A. Hungate, A. G. Abraham, L. A. Larsen, *et al.*, 2015 Staphylococcus aureus and the ecology of the nasal microbiome. Sci Adv 1: e1400216.

Lo C.-W., Y.-K. Lai, Y.-T. Liu, R. L. Gallo, and C.-M. Huang, 2011 Staphylococcus aureus hijacks a skin commensal to intensify its virulence: immunization targeting β-hemolysin and CAMP factor. J. Invest. Dermatol. 131: 401–409.

Maynard C. L., C. O. Elson, R. D. Hatton, and C. T. Weaver, 2012 Reciprocal interactions of the intestinal microbiota and immune system. Nature 489: 231–241.

Meisel J. S., G. D. Hannigan, A. S. Tyldsley, A. J. SanMiguel, B. P. Hodkinson, *et al.*, 2016 Skin Microbiome Surveys Are Strongly Influenced by Experimental Design. J. Invest. Dermatol. 136: 947–956.

Mika M., I. Mack, I. Korten, W. Qi, S. Aebi, *et al.*, 2015 Dynamics of the nasal microbiota in infancy: a prospective cohort study. J. Allergy Clin. Immunol. 135: 905–12.e11.

Nayfach S., B. Rodriguez-Mueller, N. Garud, and K. S. Pollard, 2016 An integrated metagenomics pipeline for strain profiling reveals novel patterns of bacterial transmission and biogeography. Genome Res. 26: 1612–1625.

Peterson S. W., N. C. Knox, G. R. Golding, S. D. Tyler, A. D. Tyler, *et al.*, 2016 A Study of the Infant Nasal Microbiome Development over the First Year of Life and in Relation to Their Primary Adult Caregivers Using cpn60 Universal Target (UT) as a Phylogenetic Marker. PLoS One 11: e0152493.

Proctor D. M., and D. A. Relman, 2017 The Landscape Ecology and Microbiota of the Human Nose, Mouth, and Throat. Cell Host Microbe 21: 421–432.

Rawls M., and A. K. Ellis, 2019 The microbiome of the nose. Ann. Allergy Asthma Immunol. 122: 17–24.

Reiss-Mandel A., C. Rubin, A. Maayan-Mezger, I. Novikov, H. Jaber, *et al.*, 2019 Patterns and Predictors of Staphylococcus aureus Carriage during the First Year of Life: a Longitudinal Study. J. Clin. Microbiol. 57. https://doi.org/10.1128/JCM.00282-19

Sá-Leão R., S. Nunes, A. Brito-Avô, C. R. Alves, J. A. Carriço, *et al.*, 2008 High rates of transmission of and colonization by Streptococcus pneumoniae and Haemophilus influenzae within a day care center revealed in a longitudinal study. J. Clin. Microbiol. 46: 225–234.

Scholz M., D. V. Ward, E. Pasolli, T. Tolio, M. Zolfo, *et al.*, 2016 Strain-level microbial epidemiology and population genomics from shotgun metagenomics. Nat. Methods 13: 435–438.

Shilts M. H., C. Rosas-Salazar, A. Tovchigrechko, E. K. Larkin, M. Torralba, *et al.*, 2016 Minimally Invasive Sampling Method Identifies Differences in Taxonomic Richness of Nasal Microbiomes in Young Infants Associated with Mode of Delivery. Microb. Ecol. 71: 233–242.

Song S. J., C. Lauber, E. K. Costello, C. A. Lozupone, G. Humphrey, *et al.*, 2013 Cohabiting family members share microbiota with one another and with their dogs. Elife 2: e00458.

Ta L. D. H., G. C. Yap, C. J. X. Tay, A. S. M. Lim, C.-H. Huang, *et al.*, 2018 Establishment of the nasal microbiota in the first 18 months of life: Correlation with early-onset rhinitis and wheezing. J. Allergy Clin. Immunol. 142: 86–95.

Teo S. M., D. Mok, K. Pham, M. Kusel, M. Serralha, *et al.*, 2015 The infant nasopharyngeal microbiome impacts severity of lower respiratory infection and risk of asthma development. Cell Host Microbe 17: 704–715.

Vatanen T., E. A. Franzosa, R. Schwager, S. Tripathi, T. D. Arthur, *et al.*, 2018 The human gut microbiome in early-onset type 1 diabetes from the TEDDY study. Nature 562: 589–594.

Vatanen T., D. R. Plichta, J. Somani, P. C. Münch, T. D. Arthur, *et al.*, 2019 Genomic variation and strain-specific functional adaptation in the human gut microbiome during early life. Nat Microbiol 4: 470–479.

Wollenberg M. S., J. Claesen, I. F. Escapa, K. L. Aldridge, M. A. Fischbach, *et al.*, 2014 Propionibacterium-produced coproporphyrin III induces Staphylococcus aureus aggregation and biofilm formation. MBio 5: e01286–14.

Yan M., S. J. Pamp, J. Fukuyama, P. H. Hwang, D.-Y. Cho, *et al.*, 2013 Nasal microenvironments and interspecific interactions influence nasal microbiota complexity and S. aureus carriage. Cell Host Microbe 14: 631–640.

Yassour M., E. Jason, L. J. Hogstrom, T. D. Arthur, S. Tripathi, *et al.*, 2018 Strain-Level Analysis of Mother-to-Child Bacterial Transmission during the First Few Months of Life. Cell Host Microbe 24: 146–154.e4.

Yatsunenko T., F. E. Rey, M. J. Manary, I. Trehan, M. G. Dominguez-Bello, *et al.*, 2012 Human gut microbiome viewed across age and geography. Nature 486: 222–227.

Zeevi D., T. Korem, A. Godneva, N. Bar, A. Kurilshikov, *et al.*, 2019 Structural variation in the gut microbiome associates with host health. Nature 568: 43–48.

**Second round of review**

**Reviewer #1:**

**Please provide your comments for the author(s) on the revised manuscript.**
The authors have addressed all my concerns. Congratulations on a strong manuscript!

**Has the author satisfactorily responded to your previous review?**

Yes

**Reviewer #2:**

**Please provide your comments for the author(s) on the revised manuscript.**
All my concerns and comments have been addressed. I thank the authors for a very comprehensive response!

**Has the author satisfactorily responded to your previous review?**

Yes
